# Sex-biased and parental allele-specific gene regulation by KDM6A

**DOI:** 10.1186/s13293-022-00452-0

**Published:** 2022-07-23

**Authors:** Wenxiu Ma, He Fang, Nicolas Pease, Galina N. Filippova, Christine M. Disteche, Joel B. Berletch

**Affiliations:** 1 Department of Statistics, University of California Riverside, Riverside, CA 92521 USA; 2 Department of Laboratory Medicine and Pathology, School of Medicine, University of Washington, Seattle, WA 98195 USA; 3 Department of Genome Sciences, University of Washington, Seattle, WA 98195 USA; 4 Department of Medicine, School of Medicine, University of Washington, Seattle, WA 98195 USA

**Keywords:** Sex biases, Parent-of-origin, Allele-specific, Imprinting, Gene regulation, Development, Epigenetics, Histone methylation, Chromatin

## Abstract

**Background:**

KDM6A is a demethylase encoded by a gene with female-biased expression due to escape from X inactivation. Its main role is to facilitate gene expression through removal of the repressive H3K27me3 mark, with evidence of some additional histone demethylase-independent functions. *KDM6A* mutations have been implicated in congenital disorders such as Kabuki Syndrome, as well as in sex differences in cancer.

**Methods:**

*Kdm6a* was knocked out using CRISPR/Cas9 gene editing in F1 male and female mouse embryonic stem cells (ES) derived from reciprocal crosses between C57BL6 x *Mus castaneus*. Diploid and allelic RNA-seq analyses were done to compare gene expression between wild-type and *Kdm6a* knockout (KO) clones. The effects of *Kdm6a* KO on sex-biased gene expression were investigated by comparing gene expression between male and female ES cells. Changes in H3K27me3 enrichment and chromatin accessibility at promoter regions of genes with expression changes were characterized by ChIP-seq and ATAC-seq followed by diploid and allelic analyses.

**Results:**

We report that *Kdm6a* KO in male and female embryonic stem (ES) cells derived from F1 hybrid mice cause extensive gene dysregulation, disruption of sex biases, and specific parental allele effects. Among the dysregulated genes are candidate genes that may explain abnormal developmental features of Kabuki syndrome caused by *KDM6A* mutations in human. Strikingly, *Kdm6a* knockouts result in a decrease in sex-biased expression and in preferential downregulation of the maternal alleles of a number of genes. Most promoters of dysregulated genes show concordant epigenetic changes including gain of H3K27me3 and loss of chromatin accessibility, but there was less concordance when considering allelic changes.

**Conclusions:**

Our study reveals new sex-related roles of KDM6A in the regulation of developmental genes, the maintenance of sex-biased gene expression, and the differential expression of parental alleles.

**Supplementary Information:**

The online version contains supplementary material available at 10.1186/s13293-022-00452-0.

## Highlights

- The majority of KDM6A targets are regulated from one allele or the other with allelic downregulation showing a maternal allele bias.
- *Kdm6a* knockout affects genes that have been implicated in phenotypes of Kabuki syndrome caused by *KDM6A* mutations in human.
- KDM6A facilitates sex-biased gene expression, particularly female-biased expression.

**Supplementary Information:**

The online version contains supplementary material available at 10.1186/s13293-022-00452-0.

## Introduction

Embryonic development is governed by specific patterns of epigenetic modifications to control gene expression. For example, timely activation of *Hox* gene expression via resolution of bivalency in early development is facilitated by the histone demethylase KDM6A that removes methylation at lysine 27 of histone H3 [1–4]. Activation of gene expression by KDM6A is implicated in many developmental pathways such as myogenesis, cardiac development, neural stem cell differentiation, and immune cell functions [5–9]. KDM6A can also enhance gene expression independent of its demethylase activity by association to chromatin remodeling complexes that acetylates H3K27 at active enhancers through recruitment of p300 to the MLL complex [10–12]. In addition to its major role in gene activation, evidence suggests that KDM6A can also act as a repressor of gene expression, highlighting the complexity of this protein [12, 13].

The higher level of KDM6A in female versus male mammals due to escape from X inactivation suggests a role in sex-specific gene regulation and in sex differences in health and disease [14–16]. *Kdm6a* modulates sex differences in autoimmunity and cancer, and more recently, it has been implicated in sex differences in Alzheimer’s disease [8, 17, 18]. KDM6A is encoded by a dosage-sensitive X-linked gene and has a Y-encoded homolog (UTY) that has little or no demethylase activity [19–21]. Knockout of *Kdm6a* is homozygous lethal in female mice, while a small number of runt males survive, suggesting that UTY partially compensates for loss of KDM6A possibly via demethylation-independent mechanisms [22].

We hypothesized that increased dosage of *Kdm6a* in females may lead to gene regulation of sex-biased genes and that differential expression of *Kdm6a* in male and female germline could potentially lead to parent-of-origin biased gene expression in the next generation. To address these possibilities, we investigated the role of KDM6A in sex-biased autosomal gene regulation as well as expression and epigenetic regulation of maternal and paternal alleles in mouse embryonic stem (ES) cells. *Kdm6a* knockouts (KO) were obtained by CRISPR/Cas9 approaches in ES cells derived from F1 hybrid mice from reciprocal crosses between C57BL/6 J (BL6) and *Mus castaneus* (*cast*) in which alleles can be distinguished based on SNPs. As measured by RNA-seq, sex-biased gene expression was markedly reduced following KO, with a preferential loss of female-biased expression. RNA-seq followed by allele-specific analyses revealed a subset of genes with parental allele-specific expression changes following *Kdm6a* KO, with more genes downregulated on maternal than paternal alleles.

### Methods

#### Cell lines

BC male (E14) hybrid ES cells as well as CB male hybrid ES cells derived from reciprocal crosses between C57BL/6 J and *Mus castaneus* were provided by J. Gribnau (Erasmus MC Rotterdam, NL). All ES cells were maintained in a humidified incubator at 37ºC and 5% CO_2_ in the presence of 1000 U/ml leukemia inhibitory factor (LIF) (Millipore) on a monolayer of inactivated mouse embryonic fibroblasts (MEFs) in ES media containing high glucose DMEM media supplemented with 15% fetal bovine serum (FBS), 1% non-essential amino acids, 10 mg/ml APS, 0.1 mM 2-mercaptoethanol and 25 mM L-glutamine. Medium with serum and LIF was preferred to medium containing MEK1/2 and GSK3β inhibitors (2i), which can lead to DNA methylation changes [23, 24]. Just prior to use, plates were enriched for ES cells by incubation on 0.1% gelatin coated dishes for 1 h to allow MEFs to attach, followed by transfer to fresh gelatin-coated plates for overnight culture. Following expansion, ES cells were split once (1:10) to further reduce potential MEF contamination.

### ES cell differentiation

**Fig. 1:**
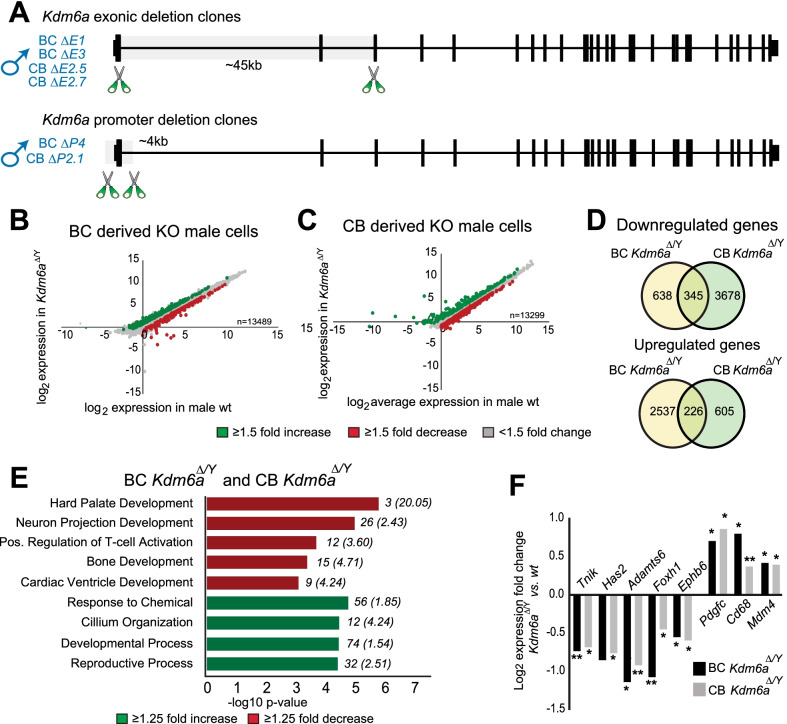
Global gene expression changes after *Kdm6a* KO in BC and CB clones. **A** Schematic illustrating the *Kdm6a* CRISPR deletions in BC and CB ES cells. Also included are clone identifiers (see Additional file 8). Exonic (*E*) deletion results in the loss of about 45 kb in length while the promoter (*P*) deletion results in a loss of about 4 kb in length. **B**, **C** Scatter plots of differential gene expression in three BC male *Kdm6a*^*Δ/Y*^ clones (*Kdm6a*^*ΔE1*^, *Kdm6a*^*ΔE3*^*,* and *Kdm6a*^*ΔP4*^) versus two male BC wt clones (**B**), and three CB male *Kdm6a *^*Δ/Y*^ clones (*Kdm6a*^*ΔE2.5*^, *Kdm6a*^*ΔE2.7*^*,* and *Kdm6a*^*ΔP2.1*^) versus two CB male wt clones (**C**). Log_2_ values of genes with > 1TPM in at least one replicate are shown. Downregulated DEGs are labeled in red, upregulated DEGs in green, and genes lacking differential expression in grey. DESeq2 was employed to identify differentially expressed genes (DEGs) in each cross using an FDR cutoff of < 0.05 and a ≥ 1.5 fold-change. **D** Venn diagrams of downregulated and upregulated genes as measured by a ≥ 1.25 fold change in log_2_ TPM in male BC and CB *Kdm6a*^*Δ/Y*^ clones versus wt. **E** GO analysis of dysregulated genes with a ≥ 1.25 fold change in log_2_ TPM found in common between the BC and CB crosses. Numbers in parentheses represent the fold enrichment over expected number of genes within a given biological process, while those not in parentheses represent the number of genes in each category. **F** Log_2_ expression fold changes for genes dysregulated in male *Kdm6a*^*Δ/Y*^ clones derived from BC and CB crosses. The genes shown are associated with processes implicated in phenotypes seen in Kabuki syndrome (*p ≤ 0.05; **p ≤ *0.01*; *Has2 p* = *.05*). See also Additional file 9

For embryoid body (EB) formation, 2 × 10^6^ wt control and *Kdm6a*^*ΔE1*^ (see Fig. 1A and Additional file 1: Fig. S1 for definition of KO clones) cells were cultured on non-adherent bacterial culture dishes without LIF for 6 days, with media changed every two days. For retinoic acid induced neuronal differentiation, 1.2 × 10^6^ wt control and *Kdm6a*^*ΔE1*^ cells were grown on gelatin-coated tissue culture plates without LIF and in the presence of 1 μM all-trans retinoic acid for 8 days, with media changed every two days. Pictures were taken at the indicated time points using a Motic AE2000 camera.

### CRISPR/Cas9 gene editing

We chose a dual sgRNA approach because of the capacity to create large deletions (Fig. 1A and Additional file 1: Fig. S1) [25]. In addition, two independent sets of sgRNAs were used to account for off target effects. First, plasmids containing sgRNAs located in exons 2 and 4 were used to delete ~ 45 kb, resulting in multiple stop codons in five of the six possible translation frames. Six-Frame translation analysis was used for in silico verification of newly acquired translation stop codons following *Kdm6a* targeting. For a second editing approach, we targeted the *Kdm6a* promoter region including exons 1 and 2. sgRNAs designed using the CHOPCHOP online tool were cloned into the CRISPR/Cas9 plasmid pX330 as described [26–28].

ES cells were transfected with sgRNAs containing CRISPR/Cas9 constructs and a plasmid carrying puromycin resistance (pPGKpuro) using UltraCruz® transfection reagent at a 3:1 CRISPR to pPGKpuro ratio in media with no antibiotics. Two days later, cells were selected for 48-72 h with 1 μg/ml puromycin in complete ES media. Cells were allowed to recover in media with antibiotics. Following recovery, cells were cloned into 96 well plates using serial dilutions. Clones were expanded and screened for deletions using PCR and Sanger sequencing. PCR analysis confirmed correct gene editing of exons 2–4 in BC male ES cell clones *Kdm6a*^*ΔE1*^ and *Kdm6a*^*ΔE3*^ and CB male ES cell clones *Kdm6a*^*ΔE2.5*^ and *Kdm6a*^*ΔE2.7*^ (Additional file 1: Fig. S1). Note that BC clones *Kdm6a*^*ΔE1*^ and *Kdm6a*^*ΔE3*^ were derived from a single CRISPR editing event. Indeed, Sanger sequencing showed that the deletion breakpoints in *Kdm6a*^*ΔE1*^ and *Kdm6a*^*ΔE3*^ were identical, suggesting that the clones were derived from a single targeting event. PCR amplification and Sanger sequencing confirmed the predicted ~ 4 kb deletion of the promoter region in BC male ES cell clones *Kdm6a*^*ΔP4*^ and CB ES clone *Kdm6a*^*ΔP2.1*^ (Additional file 1: Fig. S1).

*Kdm6a* expression in *Kdm6a*^*ΔE*^ clones was assayed by RT-PCR using primers specific for regions inside the deletion (spanning exons 3–6) and regions downstream of the deletion (spanning exons 23–26). RT-PCR and RNA-seq analyses showed low-level expression of exons downstream of the deletion, but Western blot analysis showed complete ablation of the KDM6A protein, consistent with premature stop codons and published *Kdm6a* exon KO models (Additional file 1: Fig. S1) [21, 28]. RNA-seq and RT-PCR expression analyses verified the lack of *Kdm6a* expression in clones with a promoter deletion. Note that the small alternative transcript annotated in UCSC (http://genome.ucsc.edu/) was absent in *Kdm6a*^*ΔP*^ clones as shown by the lack of reads in this region.

### Reverse-transcription quantitative PCR

RNA was extracted from cells using Qiagen RNeasy mini kit followed by cDNA synthesis with the Promega GoScript reverse transcription kit. Relative transcript levels were determined using SYBR Green PCR master mix on the ABI 7900HT machine. All qRT-PCR assays were conducted in triplicates and normalized to *Actb*; the comparative CT method was used to analyze the data.

### Western blotting

Nuclear protein lysates were prepared using the NE-PER protein extraction kit according to manufacturer’s instructions. Protein immunoblots were done to confirm protein ablation in clones *Kdm6a*^*ΔE1*^ and *Kdm6a*^*ΔE3*^. Concentrations were determined using the BCA protein assay kit. Equal concentrations of protein lysates were loaded onto SDS-PAGE gels, Tris–glycine 8%. Proteins were transferred to nitrocellulose membranes in Tris–glycine plus methanol buffer overnight at 4 °C. After blocking with Tris-buffered saline (TBS), 0.1% Tween-20, 5% nonfat dry milk for 1 h at room temperature, membranes were incubated at 4 °C overnight with the primary antibody: rabbit polyclonal antibody against KDM6A (1:3000) (Bethyl Laboratories). After primary antibody incubation, immunoblots were incubated with HRP-conjugated secondary antibodies (1:10,000) and visualized by chemiluminescence. Ponceau S staining of total protein served as a loading control.

### Sanger sequencing

All plasmid, gDNA and cDNA lysates used for Sanger sequencing were made using Qiagen PCR purification columns. All reactions were prepared and submitted according to the protocols provided by Eurofin Genomics (www.operon.com).

### RNA-seq with diploid analysis

Bulk RNA-seq indexed libraries were prepared using Illumina TruSeq RNA sample preparation kit V2. All libraries were generated from 1 µg of total RNA starting material prior to mRNA isolation. For BC derived ES cells libraries were prepared from two biological replicates from wt male controls and three *Kdm6a*^*Δ/Y*^ male clones. For CB derived ES cells libraries were prepared from two biological replicates from wt controls and three *Kdm6a*^*Δ/Y*^ clones. cDNA libraries were constructed for analysis on a NextSeq sequencer and 75 bp single-end reads were generated. Diploid gene expression was estimated using Tophat/v2.0.14 [29] with default parameters and gene-level expression was normalized using TPM (transcripts per kilobase of exon length per million mapped reads). Differentially expressed genes were determined using DESeq2 analysis [30] with a false discovery rate (FDR) threshold of 0.05 and a fold-change cutoff of 1.5. Gene ontology (GO) analysis was done using Panther Gene Ontology or DAVID GO [31–33]. Sex-biased gene expression in BC cells was determined by comparing female and male wt cells. Genes with ≥ twofold TPM expression differences (*p* ≤ 0.05; student’s *t*-test) were considered sex-biased. A gain of sex-biased expression was defined as a ≥ twofold TPM expression differences and a *p* ≤ 0.05 (student’s *t*-test) following KO. A summary of RNA-seq mapping metrics is shown in Additional file 14: Table S14. For analysis of sex-biased expression in CB cells, data from GSE90516 was re-analyzed to determine sex-biased genes in CB wt female and male cells [34]. Comparisons to CB *Kdm6a* KO cells were made following a quantile normalization.

### RNA-seq with allele-specific analysis

To estimate allele-specific gene expression in hybrid cells, a “pseudo-*castaneus*” genome was first assembled by substituting known heterozygous SNPs of *cast* (obtained from Sanger Institute 2015/May, version 5 [35]) into the BL6 mm10 reference genome. RNA-seq reads were mapped using bowtie/v2.2.5 [36] to the genome and transcriptome of both the BL6 and the pseudo-*cast* genomes. Only those reads that mapped uniquely and with a high-quality mapping score (MAPQ ≥ 30) to either the BL6 or pseudo-*cast* genomes were kept for further analyses. As previously described, all uniquely mapped and high-quality (MAPQ ≥ 30) reads were segregated into three categories: (1) BL6-SNP reads containing only BL6-specific SNP(s); (2) *cast*-SNP reads containing only *cast*-specific SNP(s); (3) allele-uncertain reads, that is, reads that do not contain valid SNPs (Additional file 14: Table S14) [37]. This resulted in ~ 11% and ~ 9% of reads mapped uniquely to the BL6 allele and the *cast* allele, respectively (Additional file 14: Table S14). For each gene $i$, denote the number of exonic BL6-SNP reads and *cast*-SNP reads be ${n}_{i0}$ and ${n}_{i1}$, respectively. The BL6-allele expression proportion for gene $i$ was then calculated as ${p}_{i0}={n}_{i0}/\left({n}_{i0}+ {n}_{i1}\right)$. To account for the mapping biases between the BL6 and the *cast* alleles, the BL6-allele expression proportion was further adjusted using the average BL6-allele mapping ratio of all autosomal genes ${r}_{m}={N}_{A0}/{N}_{A1}$, where ${N}_{A0}$ and ${N}_{A1}$ are the number of allele-specific autosomal reads in the BL6 genome and the *cast* genome, respectively. Specifically, the adjusted BL6-allele expression proportion is $\widehat{{p}_{i0}}={n}_{i0}/\left({n}_{i0}+ {{r}_{m}n}_{i1}\right)$. We assume that the diploid TPM value of gene $i$ is the sum of haploid TPM values from the BL6-allele and the *cast*-allele. Thus, the BL6-allele TPM is estimated as ${\mathrm{TPM}}_{i0}= \widehat{{p}_{i0}}{\mathrm{TPM}}_{i}$, while the *cast*-allele TPM is estimated as ${\mathrm{TPM}}_{i1}= {\left(1-\widehat{{p}_{i0}}\right)\mathrm{TPM}}_{i}$. Samples derived from the CB cross were analyzed in a similar manner with the BL6- and pseudo-*cast* reference genomes switched between the maternal and paternal alleles.

Allele-specific differential gene expression was detected using DESeq2 with similar parameters as described in the above diploid DESeq2 analysis [30]. Post filtering of allele-specific expression levels was done to remove genes with estimated allelic expression < 1TPM. Allele-specific DEGs were first identified on the maternal and paternal alleles separately, using the corresponding allelic RNA-seq read counts. Next, allelic downregulated DEGs were categorized into three groups: genes downregulated specifically on the maternal allele (Group A), genes downregulated specifically on the paternal allele (group B), and genes downregulated from both alleles (Group C). Allelic upregulated DEGs were similarly categorized into Groups D, E, and F as described in the main text. Note that our allele-specific RNA-seq analyses were performed independently from the diploid RNA-seq analyses, therefore the resulting allelic DEG list does not completely overlap with the diploid DEG list. For example, one gene could be upregulated from the maternal allele and downregulated from the paternal allele and therefore not identified as a diploid DEG.

### ATAC-seq with allele-specific analysis

ATAC-seq was done on wt male ES cells from reciprocal crosses and on clones *Kdm6a*^*ΔE1*^ and *Kdm6a*^*ΔE2.5*^ as previously described [38]. Data were generated from libraries using a NextSeq sequencer to obtain 75 bp paired-end reads. ATAC-seq reads were mapped to the BL6 genome and the pseudo-*cast* genome, separately, using bwa/ 0.7.12 [39]. Uniquely mapped reads with a high-quality mapping score (MAPQ ≥ 30) to either the BL6 or the pseudo-*cast* genome were kept for the following analyses. To assist the allele-specific analysis, ATAC-seq reads were first classified as BL6-SNP reads, *cast*-SNP reads, or allele-uncertain reads, as described in the RNA-seq analysis. As long as one-end of the paired-end read was allele-certain, then the pair was assigned to the same allele. Read pairs with two ends assigned to different alleles were discarded from further analysis. In the following text, we refer to paired-end reads simply as “reads”. Duplicate reads were removed using the MarkDuplicates function in Picard/v2.14.1. To evaluate DNA accessibility of genes transcribed on the maternal- and paternal-alleles, allelic ATAC-seq read coverage were calculated at promoter regions (2 kb upstream and downstream of TSSs). Quantile normalization was used to minimize allele biases and batch differences between cell lines derived from reciprocal crosses by assuming that the promoter read coverage has a similar distribution between alleles and cell lines.

### ChIP-seq with allelic-specific analyses

H3K27me ChIP-seq was performed on wt male ES cells from reciprocal crosses and on clones *Kdm6a*^*ΔE1*^ and *Kdm6a*^*ΔE2.5*^ as previously described [40]. ChIP libraries were prepared using the TruSeq V2 DNA preparation kit and matching input controls were sequenced by a NextSeq sequencer to produce 75 bp paired-end reads. Similar to ATAC-seq analysis (see above), ChIP-seq reads were mapped to the BL6 genome and the pseudo-*cast* genome separately, then uniquely mapped reads with high-quality mapping scores were assigned to the correct allele. Allelic promoter (± 2 kb of the TSS) coverage was scaled according to the sequencing depth and then normalized by the input control. To account for potential sequencing depth biases and allele differences, a quantile normalization method was applied (see above).

## Results

### *Kdm6a* KO in mouse ES cells results in expression changes in developmentally important genes

*Kdm6a* was edited using CRISPR/Cas9 in F1 male ES cells derived either from a cross between BL6 x *cast* (BC) or from the reciprocal cross *cast* x B6 (CB), in which alleles can be distinguished using SNPs (Fig. 1A and Additional file 1: Fig. S1) [41]. We derived stable *Kdm6a*-targeted ES cell clones from both BC and CB crosses by inducing either a deletion between exons 2 and 4 (*ΔE*) or a deletion of exons 1 and 2 in the promoter region (*ΔP*) (Fig. 1A and Additional file 1: Fig. S1A-D and Additional file 8: Table S8A). We verified editing and loss of protein in seven *Kdm6a* KO clones, including three male hemizygous clones from the BC cross, hereafter called BC *Kdm6a*^*Δ/Y*^ (if all three clones *Kdm6a*^*ΔE1*^*, Kdm6a*^*ΔE3*^, *Kdm6a*^*ΔP4*^ are included), and three male hemizygous clones from the CB cross, hereafter called CB *Kdm6a*^*Δ/Y*^ (if all three clones *Kdm6a*^*ΔE2.5*^*, Kdm6a*^*ΔE2.7*^, *Kdm6a*^*ΔP2.1*^ are included) (Fig. 1A and Additional file 1: Fig. S1A-E and Additional file 8: Table S8A). An off-target 314 kb deletion of three adjacent genes (*Sh3kbp1, Map3k15, Pdha1*) was identified in clones *Kdm6a*^*ΔE1*^ and *Kdm6a*^*ΔE3*^, consistent with a common origin of these lines (Additional file 1: Fig. S1F). These genes were excluded in DEG analysis when considering expression changes between BC wt clones and BC *Kdm6a*^*Δ/Y*^ clones. We also isolated control male ES cell clones (hereafter called CRISPR-controls) derived from the BC cross, which were subject to the CRISPR/Cas9 treatment but did not exhibit a deletion of *Kdm6a* (Additional file 8: Table S8A).

RNA-seq analysis was done to compare gene expression between wild-type (wt) and *Kdm6a* KO clones derived from the BC and CB crosses. Using principal component analysis and hierarchal clustering based on expression values for all transcribed genes, wt clones segregated from KO clones (Additional file 2: Fig. S2A, B). The CB lines clustered away from the BC lines, likely due to parental strain-specific differences including the presence of an actin-GFP transgene in the wt CB line available to us. DESeq2 was employed to identify differentially expressed genes (DEGs) in each cross using an FDR cutoff of < 0.05 and a ≥ 1.5 fold-change [30]. *Kdm6a* KO did not lead to reduced expression of pluripotency and self-renewal genes, confirming that KDM6A is not necessary to maintain pluripotency, nor did the editing process induce ES cell differentiation (Additional file 2: Fig. S2C) [42]. Consistent with previous reports, differentiation kinetics for clone *Kdm6a*^*ΔE1*^ compared to wt showed slower formation of embryoid bodies and delayed appearance of neuronal cells after retinoic acid treatment (Additional file 2: Fig. S2D) [43, 44].

In the BC cross, 195 downregulated and 334 upregulated DEGs were identified in the three male KO clones (*Kdm6a*^*Δ/Y*^) compared to the two male wt clones (Fig. 1B; Additional file 9: Table S9A). As expected, downregulated DEGs included known KDM6A targets, which were confirmed by qPCR and RT-PCR (Additional file 2: Fig. S2E, F). There did not appear to be a preference for autosomal genes (~ 4%) or sex-linked genes (~ 5%). After applying our expression fold change threshold and FDR cutoff to a previously published dataset derived from a *Kdm6a* KO in a male mouse ES line derived from an independent BC cross, we found good concordance with our results for 40% of downregulated and 31% of upregulated DEGs (Additional file 9: Table S9B) [12].

In the CB cross, we identified 334 downregulated and 213 upregulated DEGs in the three male KO *Kdm6a*^*Δ/Y*^ clones compared to two wt clones (Fig. 1C; Additional file 9: Table S9C). Again, there did not appear to be a preference for autosomal genes (~ 4%) or sex-linked genes (~ 4%). Surprisingly, the lists of DEGs differed between the CB and BC KO clones, with only 1% downregulated and 6% upregulated DEGs in common (Additional file 2: Fig. S2G and Additional file 9: Table S9A, C). Indeed, of the eight known KDM6A targets confirmed in the BC cross, only *Bcar3* and *Hsd17b11* were called as DEGs in the CB cross. This low degree of overlap was also observed when published data from a KO line from an independent BC cross was compared to the CB KO clones (11% downregulated and 8% upregulated DEGs) [12]. The discrepancy in DEGs may be due to parental strain differences. The low concordance between the CB and BC KO clones prompted us to use a less stringent approach and simply compare the lists of genes with a ≥ 1.25 fold change in TPM value between male KO and wt clones. Using this approach we identified 345 downregulated and 226 upregulated genes shared between the reciprocal crosses (Fig. 1D; Additional file 9: Table S9D, E). These genes include six known KDM6A targets including *Wt1*, *Bcar3, Foxh1, Dnmt3a, Sox3,* and *Hsd17b11*.

GO terms for downregulated genes common to BC and CB KO clones (≥ 1.25 TPM fold change) are enriched in cardiac ventricle development (e.g. *Foxh1* and *Adamts6*), consistent with heart malformations observed in utero in *Kdm6a* KO mice (Fig. 1E) [21, 43]. Interestingly, the GO terms bone development (e.g. *Has2*) and hard palate development (e.g. *Cbfb*) are also highlighted, consistent with high-arched palate and other distinctive bone and facial features observed in Kabuki type 2 syndrome caused by *KDM6A* mutations in human (Fig. 1D, E) [45–48]. There is also decreased expression of *Nes*, a KDM6A target gene identified in neural crest cells that contribute to patterning and formation of all anterior facial bone and cartilage (Additional file 9: Table S9D) [10]. Some of the downregulated genes in KO clones are associated with regulation of T-cell activation (e.g. *Ephb6*) and neuron projection development (e.g. *Tnik*), consistent with autoimmune disorders and neurological issues in Kabuki syndrome (Fig. 1E, F) [48]. Upregulated DEGs in BC and CB KO clones include a subset of genes known to be repressed by KDM6A (e.g. *Fam114a1*, *H1f0*, *Naglu*) (Additional file 9: Table S9E) [13]. Note, we did not observe increased expression of *Kdm6a’s* Y-linked paralogue, *Uty*, in any of the *Kdm6a* KO clones.

Taken together, our findings support a role for KDM6A in the regulation of a subset of key developmentally critical genes in both male and female mouse ES cells. Despite cell line and clonal variability, downregulated DEGs affirm the role of KDM6A as an enhancer of gene expression, whereas upregulated DEGs indicate a repressive function, which may reflect indirect effects on gene expression. Interestingly, some of the dysregulated genes common to the reciprocal crosses are candidates for a role in clinical features of Kabuki type 2 syndrome in human.

### KDM6A facilitates sex-biased autosomal gene expression in mouse ES cells

**Fig. 2:**
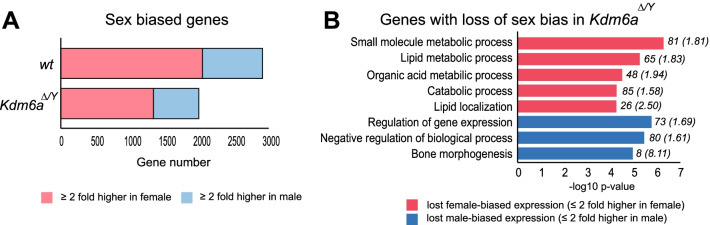
**Sex-biased gene expression changes in BC *Kdm6a* KO cells.** (**A**) Number of genes with sex-biased expression (≥ twofold TPM difference cut off; *p* < 0.05) in BC wt and *Kdm6a*^*Δ/Y*^ clones. Overall, there was a greater loss of female-biased genes (pink) than male-biased genes (blue). See also Table 1 and Additional file 10A, B. (**B**) GO analysis of genes with loss of sex-biased expression in *Kdm6a*^*Δ/Y*^. Numbers in parentheses represent the fold enrichment over expected number of genes within a given biological process, while those not in parentheses represent the number of genes in each category

Next, we investigated the role of KDM6A in regulating sex-biased autosomal gene expression. By comparing wt female and male BC ES cells we identified a 2.4 fold greater number of female-biased (2048) versus male-biased genes (868) (≥ twofold TPM difference cut off; *p* < 0.05) (Fig. 2A; Table 1; Additional file: Fig. S3A and Additional file 10: Table S10A). Interestingly, there was a marked loss of sex-biased expression in BC *Kdm6a*^*Δ/Y*^ cells (< twofold TPM difference between female wt and *Kdm6a*^*Δ/Y*^), which amounted to 35% of female-biased genes and 25% of male-biased genes (Fig. 2; Additional file 3: Fig. S3B and Additional file 10: Table S10B). To confirm these results we re-analyzed a published dataset of expression in BC wt cells and found concordance with our data for 767 sex-biased genes (Additional file 10: Table S10C) [34]. Thirty-two percent of these concordant genes showed loss of sex-biased expression following *Kdm6a* KO, which again affected more female-biased genes than male biased (Additional file 10: Table S10D). GO analysis of genes that lost female-biased expression following KO revealed several biological processes involved in lipid metabolism consistent with the role of KDM6A in adipocytes and metabolic functions (Fig. 2B) [49]. Biological processes for genes with loss of male-biased expression include bone morphogenesis, again consistent with facial features observed in Kabuki type 2 syndrome (Fig. 2B). We also observed some new sex-biased expression after *Kdm6a* KO, but at a much reduced level, with only 5% genes gaining female-biased expression and 3% genes, male-biased expression (Additional file 3: Fig. S3C, D; Table 1).

**Table 1:** Sex-biased gene expression in wt and *Kdm6a* KO clones

	wt	*Male Kdm6a*^*Δ/Y*^	
		Loss	Gain
*BC*			
Female-biased genes	2048	708 (35%)	97 (5%)
Male-biased genes	868	214 (25%)	24 (3%)
Total	2916	922 (32%)	121 (4%)
*CB*			
Female-biased genes	285	90 (32%)	
Male-biased genes	323	77 (24%)	
*Total*	608	167 (27%)	

Genes were determined to be sex-biased in wt cells based on a ≥ twofold TPM difference cut off. Wt lists the number of female- and male-biased genes in BC and CB clones. Loss and Gain indicate the number and percentage of female- and male-biased genes that were lost or gained in KO clones. For CB cells, changes in sex-biased genes were determined by comparing values from CB *Kdm6a* KO cells to wt values from published data following quantile normalization (see Methods) [34]. See also Fig. 2 and Additional file 3

In the CB cross loss of sex-biased expression after *Kdm6a* KO was examined using published data for CB male and female ES lines (Table 1; Additional file 3: Fig. S3E and Additional file 10: Fig. S10E) [34]. We found fewer sex-biased genes in wt CB cells and there was a lesser effect of *Kdm6a* KO on sex-biased gene expression in CB versus BC clones. Nonetheless, *Kdm6a* KO in CB cells again caused a greater decrease in female- (32%) than male-biased (24%) genes (Table 1; Additional file 3: Fig. S3F and Additional file 10: Fig. S10E). There was little overlap between genes that lost sex-biased expression in BC and CB clones, suggesting strain or clonal effects.

Taken together, these results suggest that KDM6A, either directly or indirectly, plays a regulatory role in perpetuating autosomal sex-biased expression in ES cells with an emphasis on maintenance of sex-biased, especially female-biased gene expression.

### The majority of gene expression changes in *Kdm6a* KO ES cells are allele-specific

To measure allelic gene expression, we assigned RNA-seq reads to parental alleles using SNPs between B6 and *cast* in the hybrid F1 mouse ES cells from the reciprocal BC and CB crosses. Most genes (94%) had informative SNPs allowing them to be classified as having either bi-allelic expression or parent-of-origin expression bias (see Methods). Allele-specific differential gene expression changes between *Kdm6a* KO and wt clones were identified using DESeq2 with the same parameters as described above (≥ 1.5 TPM fold change, FDR < 0.05). Overall, scatter plots of differential maternal and paternal expression were similar to those generated using diploid data (Additional file 4: Fig. S4A, B). However, the number of DEGs detected by allelic analysis may differ from those found by diploid analysis (see Methods).

**Fig. 3:**
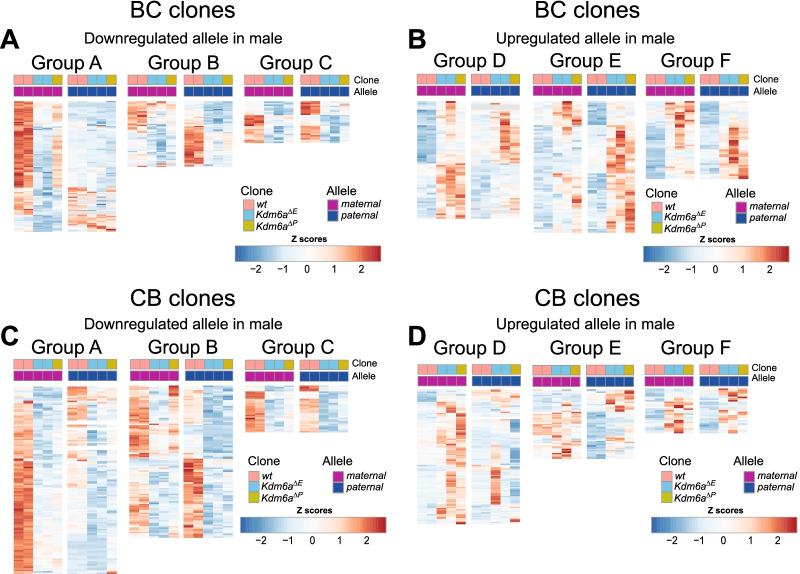
Allele-specific gene expression changes after *Kdm6a* KO in male BC and CB clones. **A**, **B** Heatmaps of allelic gene expression normalized to the mean of each gene across alleles show the extent of fold changes for maternal (purple) and paternal (dark blue) alleles of downregulated DEGs (DESeq2—≥ 1.5 fold change and FDR < 0.05) (**A**) and upregulated DEGs (**B**) in two male wt controls, three male BC KO clones *Kdm6a*^*Δ/Y*^ including two clones *Kdm6a*^*Δ/E*^ with an exon 2–4 deletion (*Kdm6a*^*ΔE1*^* Kdm6a*^*ΔE3*^) and one clone with a promoter deletion (*Kdm6a*^*ΔP4*^). The genotypes of the clones are color-coded at the top. X-linked gene expression from the paternal allele was masked as zero (white boxes). Z scores (color-coded) representing deviations from the mean for each row are shown for each group of genes. **C**, **D** Same analysis as in (**A**, **B**) for but for CB clones. See also Table 2 and Additional file 11: Table S11. Group A includes genes downregulated on the maternal allele, group B, on the paternal allele, and group C, on both alleles. Group D includes genes upregulated on the maternal allele, group E, on the paternal allele, and group F, on both alleles

Surprisingly, a majority of downregulated and upregulated DEGs displayed changes limited to one allele. In the BC cross, allelic expression analysis of *Kdm6a*^*Δ/Y*^ clones versus wt showed 167/194 (86%) downregulated and 139/189 (74%) upregulated DEGs with allele-specific changes (Table 2). Such changes were equally frequent in *Kdm6a*^*Δ/Y*^ clones from the CB cross and affected 237/269 (88%) downregulated and 171/198 (86%) upregulated genes (Table 2). However, similar to the diploid analysis above, there was little overlap between allelic DEGs in CB and BC crosses (Table 2; Additional file 11: Table S11). Downregulated DEGs in KO clones were categorized into three groups: group A includes genes downregulated on the maternal allele, group B, on the paternal allele, and group C, on both alleles (Fig. 3A, C; Table 2). Upregulated DEGs in KO clones were categorized into three groups: group D includes genes upregulated on the maternal allele, group E, on the paternal allele, and group F, on both alleles (Fig. 3B, D; Table 2).

**Table 2:** Number of allelic DEGs after *Kdm6a* KO

Allele	Group	Expression change	BC DEGs	CB DEGs	Overlap (BC and CB)
Maternal	A	Downregulated	109	133	0
Paternal	B	Downregulated	58	104	2
Biallelic	C	Downregulated	27	32	1
Total			194	269	3
Maternal	D	Upregulated	68	109	0
Paternal	E	Upregulated	71	62	1
Biallelic	F	Upregulated	50	27	0
Total			189	198	1

The number of downregulated and upregulated DEGs after *Kdm6a* KO is listed for each group (A-F, see text) at the maternal or paternal allele, at both alleles (biallelic), and the total number. BC and CB DEGs refer to differentially expressed genes between wt and *Kdm6a* KO clones from the BC and CB crosses. The number of genes that overlap between the BC and CB cells is listed in the last column. See also Fig. 3 and Additional file 11. For BC and CB cells there were 11,176 and 11,401 genes assessed from the maternal allele and 10,686 and 10,786 from the paternal allele, respectively

Remarkably, group A genes show a preferential downregulation of maternal alleles in both the BC and CB crosses. Considering the BC cross, group A comprises 109 genes, whereas group B consists of only of 58 genes (Fig. 3A; Table 2; Additional file 11: Table S11). When only considering clones with exonic *Kdm6a* deletion (*Kdm6a*^*ΔE1*^ and *Kdm6a*^*ΔE3*^) overlapping genes continue to show preferential downregulation of maternal alleles (38 for group A versus 20 for group B). Further, comparisons between individual BC KO clones show highly similar expression changes for all allelicly regulated groups (Additional file 4: Fig. S4C). In CB KO clones, there were again more group A (133) than group B genes (104). Similar to BC cells, comparisons between individual CB KO clones show highly similar expression changes for all allelicly regulated groups (Additional file 4: Fig. S4D). Despite differences between BC and CB crosses in A and B genes identified as allelic DEGs, additional analyses of changes in TPM values reveal that 33% of group A and 50% of group B genes identified in male BC clones showed a trend of decreased expression in male CB clones (Fig. 3C; Table 2; Additional file 11: Table S11 and Additional file 12: Table S12). Among downregulated genes in BC clones we found a subset of maternally (but not paternally) expressed imprinted genes that include the *Meg3* cluster*, Xlr* cluster, and *Phlda2* (Additional file 5: Fig. S5A-E and Additional file 11: Table S11A). Another maternally expressed imprinted gene *H19* that regulates *Igf2* showed a significant decrease in clones *Kdm6a*^*ΔE1*^ and *Kdm6a*^*ΔE3*^ (*p* = *0.01)* and a trend for a decrease in clone *Kdm6a*^*ΔP4*^, as well as a corresponding increase at *Igf2* (Additional file 5: Fig. S5F, G). Changes in maternally expressed imprinted genes were not verified in the CB cross where there were few changes in imprinted gene expression, suggesting clonal or parental strain effects (Additional file 5: Fig. S5H). Further comparisons between BC and CB crosses were made to address allelic expression bias of group A and B genes in wt cells. We defined allelic bias as ≥ twofold difference between alleles. We found that a majority of group A genes (maternally downregulated genes) identified in male BC clones (65%) and CB clones (57%) exhibited maternally biased expression in wt clones (Fig. 3A, C; Additional file 5: Fig. S5I, J). This maternal allele biased expression of group A genes in wt cells was also reported in an independently derived CB ES cell line [50]. Group B genes (paternally downregulated) showed a lower percentage of paternally biased expression in wt (BC—36%, CB—15%). Similar to our observations using diploid analyses, there was little overlap in the identity of genes with allelic expression biases in BC and CB wt cells, suggesting parental strain effects (Additional file 5: Fig. S5I, J).

In contrast to downregulated genes, upregulated genes did not display an allelic bias, as reflected by a similar number of genes in groups D (68) and E (71) in the male BC clones, but did display a maternal allele bias when comparing groups D (109) and E (69) in the male CB clones (Fig. 3B, D; Table 2; Additional file 11: Table S11G, H).

We conclude that KDM6A preferentially facilitates expression of maternal alleles of a subset of mouse genes. While this preference is seen in *Kdm6a* KO cells from both BC and CB crosses, the lack of overlap between the lists of genes affected in the two crosses suggests that KDM6A regulation of maternal alleles is not gene-specific. Interestingly, a majority of these genes exhibit maternal allele-biased expression in wt ES cells, suggesting that differential *Kdm6a* expression in male and female germ cells may influence parental allele expression.

### Epigenetic changes induced by *Kdm6a* KO 

**Fig. 4:**
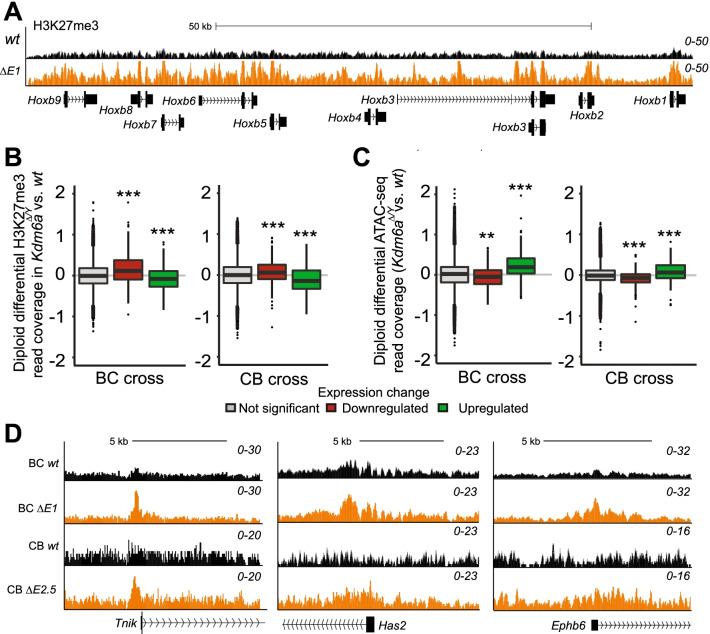
Diploid changes in H3K27me3 and chromatin accessibility after *Kdm6a* KO in BC and CB clones. **A** UCSC Genome browser (GRCm38/mm10) view of H3K27me3 profiles in wt and *Kdm6a*^*ΔE1*^ confirms a general increase across the entire *Hoxb* locus following *Kdm6a* KO. wt (black); KO cells (orange). **B** Box plots of average ratios of H3K27me3 read coverage in BC (*Kdm6a*^*ΔE1*^) and CB KO clones (*Kdm6a*^*ΔE2.5*^) versus wt at promoters (± 2 kb of the TSS) of genes with similar changes between the reciprocal crosses. Downregulated and upregulated genes exhibit expected increases and decreases in H3K27me3 (***p ≤ 0.0001). **C** Same analysis as in (**B**), but for ATAC-seq average ratios of read coverage. Downregulated and upregulated genes exhibit the expected decreases and increases in chromatin accessibility (***p ≤ 0.0001; **p = 0.011). **D** Example profiles of H3K27me3 read coverage in wt (black) and KO cells (orange) show an increase at promoters of downregulated genes (*Tnik, Has2, Ephb6*) in BC and CB KO clones. (UCSC Genome browser GRCm38/mm10)

Epigenetic changes and chromatin accessibility were examined at promoter regions (± 2 kb from the TSS) of genes with expression changes after *Kdm6a* KO, using H3K27me3 ChIP-seq and ATAC-seq to compare male BC *Kdm6a*^*ΔE1*^ and CB *Kdm6a*^*ΔE2.5*^ clones with their respective wt controls. As an initial control, we verified the expected higher H3K27me3 level at the downregulated *Hoxb* cluster in *Kdm6a* KO clones (Fig. 4A) [4, 10, 22]. Consistent with diploid gene expression changes in BC and CB clones, non-allelic analyses show most promoters of downregulated genes present with increased levels of H3K27me3 and decreased chromatin accessibility, while promoters of upregulated genes show decreased H3K27me3 and increased chromatin accessibility (Fig. 4B-D). To rule out indirect effects we verified that expression levels of genes encoding PRC2 complex-associated proteins that mediate H3K27 methylation (*Ezh1/2*, *Suz12*, *Eed*, and *Rbbp7*) were unchanged in KO clones (Additional file 6: Fig. S6A). Similarly, expression levels of genes (*Ep300*, *Smarca4*, *Eomes*) encoding chromatin remodeling proteins known to mediate H3K27 acetylation by association with KDM6A in the MLL complex were unchanged (Additional file 6: Fig. S6A). However, this analysis does not exclude changes in recruitment of these proteins.

**Fig. 5:**
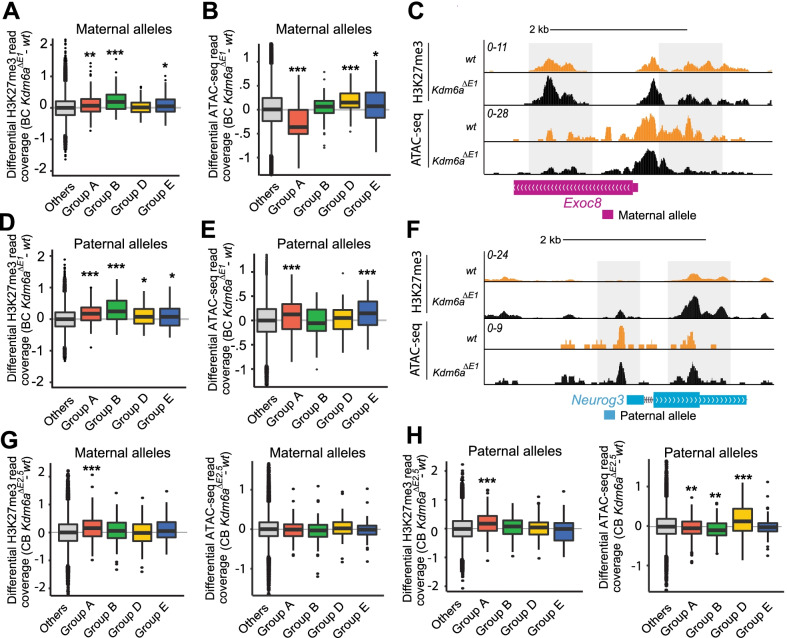
Allelic changes in H3K27me3 and chromatin accessibility after *Kdm6a* KO in BC and CB clones. **A**, **B** Box plots show average ratios of H3K27me3 (**A**) and ATAC-seq (**B**) read coverage between clone *Kdm6a*^*ΔE1*^ and wt cells at promoters (± 2 kb of the TSS) on maternal alleles. Both downregulated groups (**A** and **B**) show a significant increase in H3K27me3 levels on maternal alleles (**A** **p = *0.001;* **B** ***p = *5.33*^*e−06*^). Maternally downregulated genes (group A) show significant loss of chromatin accessibility (***p = *5.9*^*e−11*^) on the maternal allele in KO cells, while paternally downregulated genes (group B) do not. Upregulated groups (**D** and **E**) show no or slight changes in H3K27me3 (**E** *p = *0.020*), and show the expected increase in chromatin accessibility (**D** ***p = *2.4*^*e−07*^; **E** *p = *0.03*). Values were normalized to the input and converted to log scale. **C** UCSC Genome browser (GRCm38/mm10) view of maternal H3K27me3 and chromatin accessibility profiles in wt (−) and *Kdm6a*^*ΔE1*^ ( +) at the maternally downregulated gene *Exoc8* shows an increase in H3K27me3 and a corresponding decrease in ATAC-seq around the promoter. **D**, **E** Same analysis as in (**A**, **B**) but for paternal alleles. Both downregulated groups (A and B) show an increase in H3K27me3 levels (**A** ***p = *4.32*^*e−07*^*;* **B** ***p = *1.73*^*e−06*^), while upregulated groups (**D** and **E**) show a slight but significant increase (**D** *p = *0.03;* **E** *p = *0.04*)*.* Only maternally downregulated group A and paternally upregulated group E show significant changes in ATAC-seq (**A** ***p = *0.0001*; **B** ***p = *0.001*) at paternal alleles*.* **F** UCSC Genome browser (GRCm38/mm10) view of paternal H3K27me3 and chromatin accessibility profiles in wt (−) and *Kdm6a*^*ΔE1*^ ( +) at the paternally downregulated *Neurog3* gene shows increased H3K27me3 but no corresponding decrease in ATAC-seq around the promoter. **G** Same analysis as in (**A**, **B**) but in the CB *Kdm6a*^*ΔE2.5*^ clone. Only maternally downregulated genes (group A) show increased H3K27me3. There were no significant ATAC-seq changes. **H** Same analysis as in (**D**, **E**) but in the CB *Kdm6a*^*ΔE2.5*^ clone. Maternally downregulated group A show increased H3K27me3, while ATAC-seq changes were only seen on paternal alleles. Group A includes genes downregulated on the maternal allele and group B on the paternal allele. Group D includes genes upregulated on the maternal allele and group E, on the paternal allele

Next, we performed allele-specific ChIP-seq and ATAC-seq to detect H3K27me3 and chromatin accessibility changes on each parental allele. In BC KO cells a significant increase in H3K27me3 at the promoters of maternally downregulated genes (group A) was accompanied by a significant decrease in chromatin accessibility, while maternally upregulated group D and E genes showed the expected increase in chromatin accessibility (Fig. 5A-C; Additional file 7: Fig. S7A and Additional file 13: Table S13). In contrast, paternally downregulated alleles (group B) showed an increase in H3K27me3 but no significant decrease in chromatin accessibility, while paternally upregulated (group E) genes showed the expected increase in chromatin accessibility (Fig. 5D-F; Additional file 7: Fig. S7A and Additional file 13: Table S13). Importantly, in clones with exonic *Kdm6a* deletion (*Kdm6a*^*ΔE1*^ and *Kdm6a*^*ΔE3*^), promoters of overlapping genes show similar changes in H3K27me3 and chromatin accessibility (Figs. 3A, B; Additional file 7: Fig. S7B, D, F, H). In the CB cross similar findings for H3K27me3 were obtained for group A genes, while upregulated groups D and E genes showed only a modest increase in chromatin accessibility (Fig. 5G, H; Additional file 13: Table S13). Of note, differences in chromatin accessibility were only observed at promoters of KDM6A target genes, and there were no overall differences in accessibility at other promoters or enhancers (Additional file 6: Fig. S6B, C). Additionally, H3K27me3 showed no overall differences at enhancers regardless of parental allele (Additional file 6: Fig. S6).

Taken together our results show the expected changes in H3K27me3 and chromatin accessibility at upregulated and downregulated DEGs. Allelic analysis demonstrates parental allele-specific changes, notably for downregulated group A genes, which display a consistent increase in H3K27me3 on both parental alleles in both crosses. However, chromatin accessibility changes were partly inconsistent with changes in H3K27me3 in BC and CB KO clones, a phenomenon observed in other studies [10, 51, 52].

## Discussion

Here, we show that *Kdm6a* KO in ES cells derived from reciprocal crosses between C57BL/6 J and *Mus castaneus* results in significant dysregulation of genes involved in development and reproduction. Depletion of KDM6A leads to a loss in sex-biased gene expression, particularly female-biased expression, suggesting a new role for the histone demethylase. We also found a strong downregulation of maternal alleles in *Kdm6a* KO clones from both reciprocal crosses. Furthermore, a large subset of the target genes exhibits maternal allele expression bias in wt ES cells from both crosses. The higher expression of *Kdm6a* in females versus males due to escape from X inactivation may explain the preferential regulation of maternal allele expression.

Gene regulation by KDM6A mainly depends on its catalytic function. Indeed, depletion or inhibition of KDM6A results in decreased gene expression due to higher levels of the repressive histone mark H3K27me3 at promoters and/or enhancers of target genes, which readily explains our findings of H3K27me3 enrichment and downregulation of genes after *Kdm6a* KO [14, 42, 53]. Conversely, higher *KDM6A* expression in human cancer causes a decrease of H3K27me3 enriched heterochromatin [54]. Aside from its catalytic function, KDM6A can also enhance gene expression through other mechanisms that involve recruitment of the MLL complex to increase histone acetylation [10–12]. Finally, KDM6A may act as a repressor of gene expression, underscoring the complexity of this protein [10, 11, 13, 55]. Indirect effects are also expected, which could explain some of our findings of upregulated genes after *Kdm6a* KO. In particular, the more than twofold reduction in *Dnmt3a* expression in KO male BC clones could cause DNA hypomethylation and upregulation of DNMT3A target genes, such as *Celsr1* and *Fah* [56]. Similarly, the decrease in *Tet1* expression in KO female ES cells could explain increased expression of TET1 target genes such as *Casp8*.

A number of KO dysregulated genes were found to differ between crosses, which could be due to differences between the wt ES cell lines and/or to strain differences. Clonal variability is a limitation of the study, which we addressed by using both a stringent DESeq2 approach and a less stringent fold change approach to identify gene expression changes in BC and CB KO cells. Importantly, among the KDM6A targets common to both reciprocal crosses we identified a subset of genes associated with multiple phenotypes observed in individuals with Kabuki syndrome due to mutations in *KDM6A* in human. For example, *Has2* and *Cbfb* have been implicated in hard palate development, malformations of which are often seen in Kabuki syndrome [48, 57, 58]. *Ephb6* and *Tnik* were also downregulated in *Kdm6a* KO cells derived from reciprocal crosses consistent with immunopathological phenotypes and neurological disorders seen in the syndrome [48]. Our identification of KO dysregulated genes involved in development, immune cell functions, neuronal cell functions, and metabolism may help further identify KDM6A targets implicated in Kabuki syndrome, a disorder with more severe phenotypes in males [48, 59, 60].

We report on a new role for KDM6A, which is to maintain sex-biased, especially female-biased gene expression. KDM6A appears to maintain female-biased expression via repression of genes in males, which is surprising since KDM6A’s main function is to facilitate gene expression through removal of H3K27me3. However, this may be due to indirect effects of KDM6A depletion as mentioned above. Indeed, there appears to be little commonality in genes that gain sex bias following KO.

A surprising finding from our allelic expression analyses is that a majority of gene dysregulation after *Kdm6a* KO occurs on one allele or the other, with a strong maternal allele bias. This novel observation was made in both reciprocal crosses, even though the sets of dysregulated alleles differ. Female-biased expression of *Kdm6a* due to escape from X inactivation is consistent with a role in differential regulation of the maternal and paternal genomes [61]. How this allelic regulation is transmitted through generations will need to be further investigated. H3K27me3 is known to play a critical role in the repression of the maternal allele at non-canonical imprinted sites, loss of which leads to loss of imprinting [62]. Thus, manipulating the dosage of *Kdm6a* in differentiating hybrid primordial germ cells where genomic imprints are established may yield insights into its role in regulating non-canonical imprinted genes. Further, utilizing independent reciprocal crosses with *Kdm6a* KO will help to identify potential non-canonical imprinted gene targets. Future allelic expression studies using differentiating ES cells following KO would be interesting since KDM6A target genes may also have roles in germ layer development and ES cell differentiation [14, 17]. We found changes in H3K27me3 and chromatin accessibility at dysregulated alleles but these were not always concordant with expression changes. This was not entirely unexpected as H3K27me3 levels are not always correlated with expression [10, 52]. Furthermore, many of allelicly repressed genes were more highly expressed from downregulated alleles in wt suggesting a higher sensitivity to changes in H3K27me3.

### Perspectives and significance

Results from our study provide important findings implicating KDM6A, a female-biased chromatin modifier, in allelic gene regulation with a preference for maternal alleles. These data emphasize that inclusion of allelic analyses of gene regulation will enhance our understanding of parent-of-origin effects and sex-specific differences. Our study also provides evidence of a role of KDM6A in the regulation of sex-biased gene expression, which could have broad implications in understanding sex differences. Our CRISPR deletions encompass known pathogenic mutations seen in Kabuki syndrome, including loss of exons 1–2 [63]. Thus, results from this study may reveal therapeutic targets for individuals with Kabuki syndrome.

Considering KDM6A’s role in embryonic development, it will be of interest to determine whether sex-biased and parental allele effects of depletion persist during differentiation. *Kdm6a* KO mice have a sex-specific phenotype, with male mice runt but able to survive, while female mice die during embryogenesis [21]. This has been attributed to a demethylase-independent mechanism of compensation by UTY, which has little or no demethylase activity [21]. A number of escape genes have a Y-linked paralogues that may have synergistic roles in gene regulation. Therefore, it will be important to fully tease out the possible compensatory role of *Uty* on gene expression by generating ES cell clones with *Kdm6a* and *Uty* specific KO. Future analyses considering additional epigenetic marks such as H3K4me3, H3K9ac and DNA methylation will help to ascertain the complex network of gene regulation by KDM6A. Finally, extending our studies of KDM6A’s role in gene regulation to human cells will help determine whether KDM6A’s functions are conserved between mice and humans at target genes and regulatory elements.

## Conclusions

Here, we demonstrate for the first time a role in the regulation of sex-biased genes and a maternal allele bias in gene regulation by a gene that escapes X inactivation, a uniquely female phenomenon. Our results implicate KDM6A as a master regulator of fetal growth and development through the control of a subset of maternally biased genes. While our studies are performed in ES cells where developmental pathways are not fully represented, our data provide information on pathways and lineages that may be impacted in syndromes due to *KDM6A* mutations such as Kabuki Syndrome.

## Supplementary Information

**Additional file 1: Fig. S1.** CRISPR/Cas9 editing of *Kdm6a* in BC and CB mouse ES cells. (**A**) Schematic shows location of the exonic deletion (*Kdm6a*^*ΔE*^) that removed exons 2–4 of *Kdm6a* in male BC and CB ES cells. Exons are shown as vertical bars with location of the PCR and RT-PCR primers (color-coded arrows) used to confirm the deletion and measure expression. Above, images of gels after electrophoresis of PCR products (gDNA), RT-PCR products (cDNA), and Western blot (protein) confirm KDM6A KO in male BC clones (*Kdm6a*^*ΔE1*^*, Kdm6a*^*ΔE3*^) and CB clones (*Kdm6a*^*ΔE2.5*^*, Kdm6a*^*ΔE2.7*^) compared to wt (color-coding refers to primers shown on schematic). *Actb* was run as a control for PCR and RT-PCR and Ponceau S staining was used as loading control for the Western blot. Sanger sequencing shown below the schematic was done to verify deletions in all male *Kdm6a*^*ΔE*^ clones compared to wt. (**B**) Schematic shows location of the promoter region deletion (*Kdm6a*^*ΔP*^) made in male BC and CB ES cells. Exons are shown as vertical bars with location of the PCR and RT-PCR primers (color-coded arrows) used to confirm the deletion and measure expression. Above, images of gels after electrophoresis of PCR products (gDNA) and RT-PCR products (cDNA) confirm KDM6A KO in male BC clone (*Kdm6a*^*ΔP4*^) and CB clone (*Kdm6a*^*ΔP2.1*^) compared to wt. *Actb* was run as a control for PCR and RT-PCR. Sanger sequencing shown below the schematic was done to verify deletions in all *Kdm6a*^*ΔP*^ male BC and CB clones. (**C-D**) UCSC genome browser (GRCm38/mm10) view of RNA-seq profiles for (**C**) BC male and female wt, BC male clones *Kdm6a*^*ΔE*^ and *Kdm6a*^*ΔP*^, and (**D**) CB male wt, and CB male *Kdm6a*^*ΔE*^ and *Kdm6a*^*ΔP*^ clones*.* Note that a low level of 3’ end reads are present in clones with deletion of exons 2–4, although there is no evidence of protein (see A above). In clones with deletion of the promoter, there are no reads over *Kdm6a*, including no reads overlapping the small alternative *Kdm6a* transcript, confirming absence of expression and suggesting the absence of a cryptic promoter for the smaller annotated transcript. *Actb* was used as a PCR control. Primer sequences and sgRNAs are listed in Additional file 8. (**E**) Evidence of an off-target event in male BC clones *Kdm6a*^*ΔE1*^ and *Kdm6a*^*ΔE3*^. RNA-seq genome browser profiles in BC derived *wt* and *Kdm6a* KO male clones show absence of *Pdha1* reads in *Kdm6a*^*ΔE*^ but not *Kdm6a*^*ΔP*^ clones. The inset shows PCR analysis for regions A, B and C amplified by primers indicated by colored arrows, which confirms an off-target deletion of part of *Sh3kbp1* and all of *Map3k15* and *Pdha1* in BC *Kdm6a*^*ΔE*^ clones. No other KO clones show this off-target deletion. *Actb* was run as a control.

**Additional file 2: Fig. S2.** Diploid gene expression changes in *Kdm6a* KO BC and CB clones. (A) Principal component analysis (PCA) based on diploid expression values for all transcribed genes from RNA-seq in wt and *Kdm6a* KO clones derived from the BC and CB crosses. BC clones include two male wt, two female wt, three male KO (*Kdm6a*^*Δ/Y*^), while CB clones include two male wt and three male KO (*Kdm6a*^*Δ/Y*^). Clone identifiers are included in the plot. (B) Hierarchal clustering of the clones described in (A). The color scale represents the sample-to-sample distance. (C) Expression fold changes between BC derived *Kdm6a*^*Δ/Y*^ clones (*Kdm6a*^*ΔE1*^, *Kdm6a*^*ΔE3*^*,* and *Kdm6a*^*ΔP4*^) and wt measured by RNA-seq show a significant decrease in *Kdm6a* expression, but no significant decrease in expression of pluripotent genes (*Nanog, Pou5f1, Sox2, Cd9, Stat3, Fut4*) (**p < *0.01* using a student’s *t*-test). (D) Deficiencies in ES cell differentiation potential following *Kdm6a* KO were tested by removal of LIF and by *all*-*trans* retinoic acid (RA) treatment. Smaller and less dense embryoid bodies were observed 6 days after removal of LIF in *Kdm6a*^*ΔE1*^. In the presence of RA, wt cells show morphological signs of differentiation after 8 days, while *Kdm6a*^*ΔE1*^ cells remain similar in morphology to day 0 controls. (E) Expression fold changes between male BC *Kdm6a*^*Δ/Y*^ clones (*Kdm6a*^*ΔE1*^, *Kdm6a*^*ΔE3*^*,* and *Kdm6a*^*ΔP4*^) and wt measured by RNA-seq confirm decreased expression of known KDM6A target genes *Wt1, Bcar3, Foxh1, Dnmt3a, Sox3, Hsd17b11*. Expression is based on diploid analysis at **FDR < 0.001. (F) Expression fold changes between male BC *Kdm6a*^*Δ/Y*^ clones (*Kdm6a*^*ΔE1*^, *Kdm6a*^*ΔE3*^*,* and *Kdm6a*^*ΔP4*^) and wt measured by quantitative RT-PCR analysis confirm downregulation of *Vrtn*, *Bcar3*, and *Sall2* (*p < 0.01 using a student’s *t*-test) (Additional file 9A). Expression is normalized to *Actb*. (**G**) Venn diagrams to compare the number of downregulated and upregulated DEGs in male BC *Kdm6a*^*Δ/Y*^ clones (*Kdm6a*^*ΔE1*^, *Kdm6a*^*ΔE3*^*,* and *Kdm6a*^*ΔP4*^) and male CB *Kdm6a*^*Δ/Y*^ clones (*Kdm6a*^*ΔE2.5*^*, Kdm6a*^*ΔE2.7*^, *Kdm6a*^*ΔP2.1*^). DESeq2 was employed to identify differentially expressed genes (DEGs) in each cross using an FDR cutoff of < 0.05 and a ≥ 1.5 fold-change.

**Additional file 3: Fig. S3.** Changes in sex-biased gene expression in *Kdm6a* KO BC and CB clones. (A) Scatter plots of log_2_ gene expression between male and female lines illustrating the degree of sex-biased expression in wt cells. Sex-biased genes were identified by comparing two BC male wt clones versus two BC female male wt clones. A gene was classified as sex-biased if its expression is ≥ 2 TPM fold expression higher in either female wt or male wt cells (*p* ≤ 0.05). Female-biased genes are in light orange and male-biased genes in light blue. (B) Scatter plot showing loss of sex-biased expression in three BC male *Kdm6a*^*Δ/Y*^ clones (*Kdm6a*^*ΔE1*^, *Kdm6a*^*ΔE3*^*,* and *Kdm6a*^*ΔP4*^) versus two BC female wt clones. Genes that lost sex-biased expression in KO cells are in dark orange if female-biased in wt, and in dark blue if male-biased in wt. (C) Scatter plot of genes without sex-biased expression (< 2 TPM fold expression difference) in wt BC cells. (D) Gain of sex biased expression in in three BC male *Kdm6a*^*Δ/Y*^ clones (*Kdm6a*^*ΔE1*^, *Kdm6a*^*ΔE3*^*,* and *Kdm6a*^*ΔP4*^). (E, F). Scatter plots of log_2_ gene expression of sex-biased genes in (E) three CB male wt clones versus three CB female wt clones, (F) three CB male *Kdm6a*^*Δ/Y*^ (*Kdm6a*^*ΔE2.5*^, *Kdm6a*^*ΔE2.7*^*,* and *Kdm6a*^*ΔP2.1*^) clones versus three CB female wt clones. Same analysis as in A, B. All wt expression values are from re-analysis of published RNA-seq data in CB ES cells, as we did not have access to a female wt CB line [34]. See also Table 1 and Additional file 10: Table S10.

**Additional file 4: Fig. S4.** Correlation of gene expression (diploid, maternal and paternal alleles) between wt and KO clones. Scatter plots of average gene expression based on TPM values show a high correlation between male BC wt and (A) *Kdm6a*^*Δ/Y*^ clones (*Kdm6a*^*ΔE1*^, *Kdm6a*^*ΔE3*^*,* and *Kdm6a*^*ΔP4*^), and (B) male CB *Kdm6a*^*Δ/Y*^ clones (*Kdm6a*^*ΔE2.5*^, *Kdm6a*^*ΔE2.7*^*,* and *Kdm6a*^*ΔP2.1*^) for diploid and allele-specific expression. Correlation coefficients are all ≥ 0.96. (C, D) Scatter plots of diploid TPM expression values comparing individual BC KO clones (C) and CB KO clones (D). Red dots represent all allelicly regulated genes (combined groups A-F). Black dots represent all other genes. High correlation coefficients (c.c) indicate similar expression changes following KO in all three clones.

**Additional file 5: Fig. S5.** A subset of maternally expressed imprinted genes are downregulated after *Kdm6a* KO in BC but not in CB clones. (A) Scatter plot of average expression changes for canonical imprinted genes, either maternally (purple) or paternally (blue) expressed, in *Kdm6a*^*Δ/Y*^ (*Kdm6a*^*ΔE1*^, *Kdm6a*^*ΔE3*^*,* and *Kdm6a*^*ΔP4*^) versus wt (average from 2 clones) based on diploid RNA-seq analysis in male BC derived clones. Only imprinted genes with > 1TPM in at least one sample are shown. (B) RT-PCR analysis confirms decreased expression of *Meg3* and *Rian* in all male BC KO clones (*Kdm6a*^*ΔE1*^*, Kdm6a*^*ΔE3*^, *Kdm6a*^*ΔP4*^). CRISPR + /– labels indicate presence/absence of transfection with CRISPR/Cas9 and *Kdm6a* sgRNAs, while a ± for *Kdm6a*^*ΔP*^ and *Kdm6a*^*ΔE*^ clones indicates editing result. Non-edited CRISPR control clones (*Kdm6a*^*E14*^*, Kdm6a*^*P2*^) maintain expression of these genes. (C) Sanger sequencing in wt male BC clones confirmed the presence of SNPs (gDNA) and mono-allelic expression (cDNA) of *Meg3* and *Rian*. (D, E) UCSC Genome browser (GRCm38/mm10) views of allele-specific RNA-seq profiles at the maternally expressed *Dlk1*/*Mirg* polycistron region and at a subset of imprinted *Xlr* genes. Allelic read profiles on the maternal chromosome (purple) and the paternal chromosome (blue) are based on SNP analysis in male BC *wt* clone (KO-), compared to male BC *Kdm6a*^*ΔE1*^ clone (KO +)*.* (F) UCSC Genome browser (GRCm38/mm10) view of allele-specific RNA-seq, ATAC-seq and H3K27me3 ChIP-seq profiles generated in male *wt* and *Kdm6a*^*ΔE1*^ at the imprinted genes *H19* (maternally expressed) and *Igf2* (paternally expressed). The DMR (differentially methylated region) is denoted by a green bar. (G) TPM expression fold change measured by RNA-seq for *H19* and *Igf2* in *Kdm6A*^*ΔE1*^ versus wt shows *H19* downregulation concordant to the expected upregulation of *Igf2*. (H) Same analysis as in (A) for male CB *Kdm6a *^*Δ/Y*^ clones (*Kdm6a*^*ΔE2.5*^, *Kdm6a*^*ΔE2.7*^*,* and *Kdm6a*^*ΔP2.1*^). (I, J) Venn diagrams to compare the number of allele-biased genes in BC male wt, BC female wt, and CB male wt. Allelic bias was defined as a ≥ twofold difference between alleles. BC derived cells show more commonality in allele-biased genes than with CB derived cells.

**Additional file 6: Fig. S6.** Chromatin modifier expression and chromatin accessibility changes at enhancers and promoters after *Kdm6a* KO. (A) Expression fold changes between *Kdm6a*^*Δ/Y*^ clones (*Kdm6a*^*ΔE1*^, *Kdm6a*^*ΔE3*^*,* and *Kdm6a*^*ΔP4*^) and wt measured by RNA-seq for genes encoding known chromatin modifying proteins in the PRC2 complex (*Ezh1/2, Suz12, Eed, Rbbp7*) and the MLL complex (*Ep300, Smarca4, Eomes*). (B) Box plots of allelic ATAC-seq read coverage at gene enhancers in male BC and CB clones (*Kdm6a*^*ΔE1*^ and *Kdm6a*^*ΔE2.5*^) compared to their respective wt controls. Enhancers (*n* = 25,346) were defined as regions enriched in H3K4me3 and H3K27ac in male ES cells as described [12]. (C) Same analysis as in (B) but for promoters (*n* = 20,745). ATAC-seq coverage was calculated using ± 2 kb surrounding the transcription start sites determined using GENCODE. No significant chromatin accessibility differences were seen, nor was there a significant difference between alleles in either cross. (D) Same analysis as is (B) but for H3K27me3.

**Additional file 7: Fig. S7.** Allelic epigenetic features of genes regulated by KDM6A. (A) Scatter plots of H3K27me3 read coverage in log_2_ scale on maternal and paternal alleles in male BC *Kdm6a*^*ΔE1*^ cells versus wt. Allele-specific ChIP-seq reads around promoters (± 2 kb of the TSS) were used to calculate promoter coverage normalized by sequencing depth and allele differences (see also Fig. 5A, D). (B) Same analysis as in (A) but for DEGs that overlap between exonic KO clones only (*Kdm6a*^*ΔE1*^ and *Kdm6a*^*ΔE3*^) and *Kdm6a*^*ΔY*^ clones (*Kdm6a*^*ΔE1*^*, Kdm6a*^*ΔE3*^ and *Kdm6a*^*ΔP4*^). (C) Scatter plots of ATAC-seq read coverage in log_2_ scale on maternal and paternal alleles in male BC *Kdm6a*^*ΔE1*^ cells versus wt. Allele-specific reads around promoters (± 2 kb of the TSS) were used to calculate promoter coverage normalized by sequencing depth and allele differences (see also Fig. 5B, E). (D) Same analysis as in (C) but for DEGs that overlap between exonic KO clones only (*Kdm6a*^*ΔE1*^ and *Kdm6a*^*ΔE3*^) and *Kdm6a*^*ΔY*^ clones (*Kdm6a*^*ΔE1*^*, Kdm6a*^*ΔE3*^ and *Kdm6a*^*ΔP4*^). (E, F) Same analysis as in (A) and (B), but for upregulated DEGs. (G, H) same analysis as in (C) and (D), but for upregulated DEGs. (I, J) Scatter plots of ratios of ATAC-seq read coverage versus ratios of H3K27me3 ChIP-seq read coverage between CB clone *Kdm6a*^*ΔE2.5*^ and wt CB cells at promoter regions (± 2 kb of the TSS) of downregulated groups on maternal and paternal alleles. (K, L) Same analysis as in (I) and (J) but for upregulated groups. See also Fig. 5G, H. Unedited image figure legend. (A) Raw images for electrophoresis gels shown in Additional file 1A for BC and CB clones. Left panel shows screening of CRISPR exon deletion clones by PCR. The top right gel shows screening of CRISPR exon deletion clones in CB cells by PCR (gDNA). The associated control *Actb* lanes are in (**C**) lower right gel (gDNA). For all panels, primer names correspond to those in Additional file 1A. (**B**) Unedited images of western blots and controls for KDM6A. Top two are film images after 30 s and 5 min exposure (30 s image is shown in Additional file 1A). Lower image shows a picture of the Ponceau S stain used for loading control. For all images the clones are labelled at the top. (**C**) Raw gels for *Kdm6a* expression changes in exon deletion clones. Left panel shows *Kdm6a* expression changes in BC clones. The center panel shows a repeat electrophoresis run of the *Actb* controls which is shown in Additional file 1A. For CB clones, *Kdm6a* expression in controls and KO cells are shown in the lower right gel in (**A**) (cDNA). The right panel shows the *Actb* control (cDNA). Primer names correspond to those in Additional file 1A. (**D**) Raw images for electrophoresis gels shown in Additional file 1B for BC and CB clones. Left panel shows promoter deletion screening by PCR in BC clones (gDNA). The center and right panels show the same for the CB promoter clone (gDNA). For all panels, primer names correspond to those in Additional file 1B. (**E**) Raw gels for *Kdm6a* expression changes in control and promoter deletion clones. Top gel shows expression changes in BC KO clones and the lower gel shows expression changes in the CB KO clone. Primer names correspond to those in Additional file 1B. (**F**) Gel image of PCR screening for the off-target effect seen in CRISPR KO BC exon clones ΔE1, and ΔE3. Primer names correspond to those in Additional file 1F. (**G**) Gel images showing expression changes of *Meg3* (left) and *Rian* (center) in controls and BC KO clones. The right panel shows the *Actb* controls.

**Additional file 8: Table S8.** A: Summary of clones, B: Oligos used for CRISPR and PCR.

**Additional file 9: Table S9.** A: Expression changes in BC Kdm6aΔY clones (see Figure 1B); B: GSE97702 comparison; C: Expression changes in CB Kdm6aΔY KO clones (see Figure 1C); D: Downregulated genes common between BC and CB KO clones (see Figure 1D-F); E: Upregulated genes common between BC and CB KO clones (see Figure 1D-F).

**Additional file 10: Table S10.** A: Sex-biased genes in wt ES cells (see Figures 2A and Additional file 3A); B: Loss of sex-biased expression in Kdm6aΔY ES cells (see Figures 2 and Additional file 3B); C: Concordant sex-biased genes (Werner et al. 2017); D: Loss of sex-biased expression in Kdm6aΔY for concordant genes; E: Loss of sex-biased expression in CB Kdm6aΔY clones (see Additional file 3F).

**Additional file 11: Table S11.** A: BC group A (see Figures 3A, B); B: BC group B (see Figures 3A, B); C: BC group D (see Figures 3A, B); D: BC group E (see Figures 3A, B); E: CB group A (see Figures 3C, D); F: CB group B (see Figures 3C, D); G: CB group D (see Figures 3C, D); H: CB group E (see Figures 3C, D).

**Additional file 12: Fig. S12.** A: Expression of BC Group A genes in CB clones; B: Expression of BC Group B genes in CB clones.

**Additional file 13: Fig. S13.** A: BC H3K27me3 ChIP-seq changes (see Figures 5 and Additional file 7); B: BC ATAC-seq changes (see Figures 5 and Additional file 7); C: CB H3K27me3 changes (see Figures 5 and Additional file 7); D: CB ATAC-seq changes (see Figures 5 and Additional file 7).

**Additional file 14: Fig. S14.** Summary of RNA-seq mapping.

## Data Availability

The datasets generated and/or analyzed during the current study are available in the NCBI GEO database. Oligonucleotide sequences used in this study are available in Additional file 1.
