# Supplementary figures and images for: Sex-biased and parental allele-specific gene regulation by KDM6A
Source: Biol Sex Differ. 2022 Jul 23;13:40. doi: 10.1186/s13293-022-00452-0 (PMC9308343; doi:10.1186/s13293-022-00452-0)

Figure S1

**A**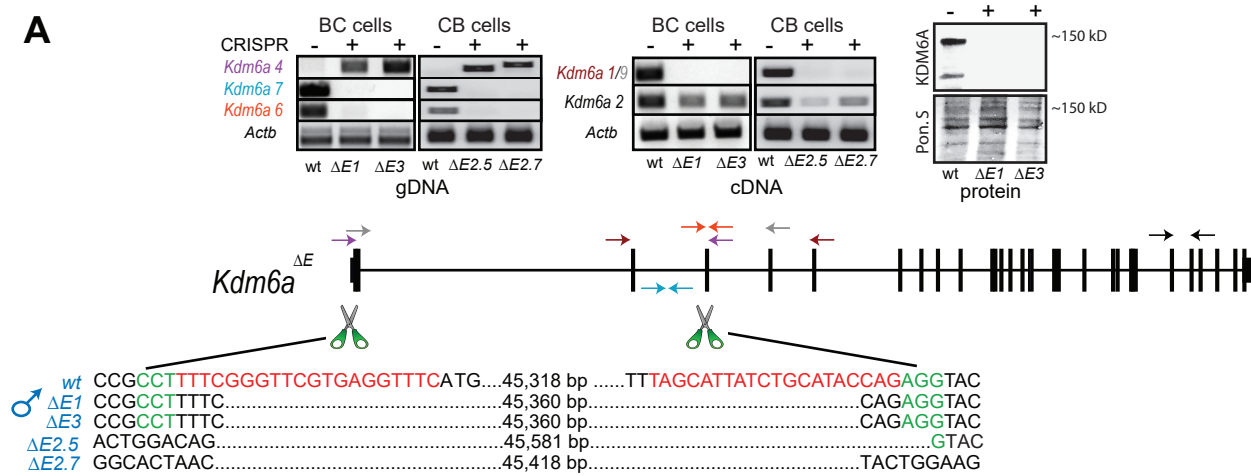**B**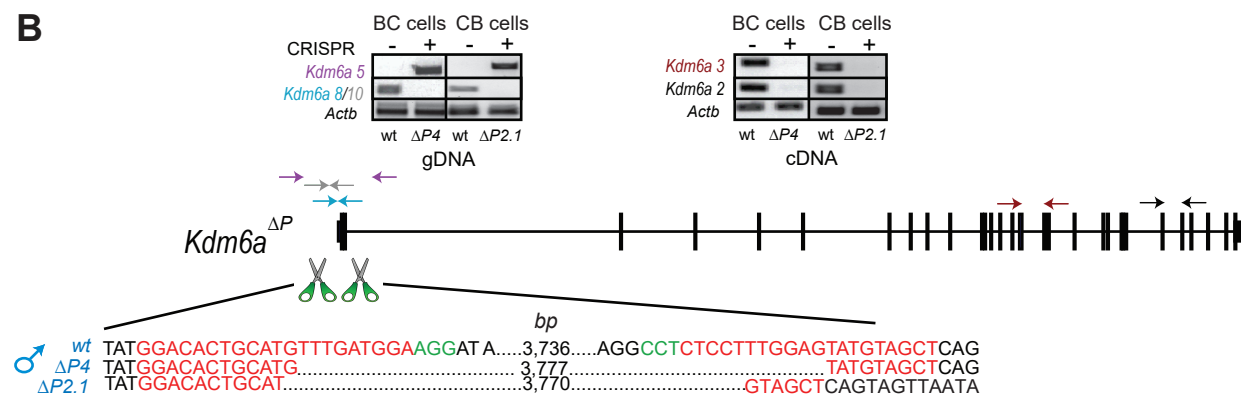**C**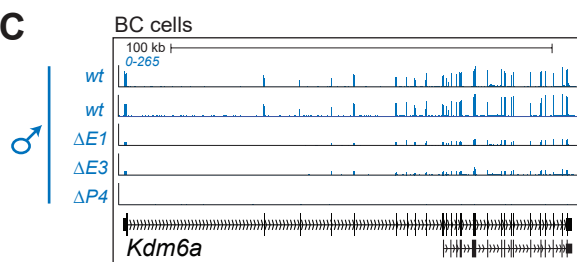**D**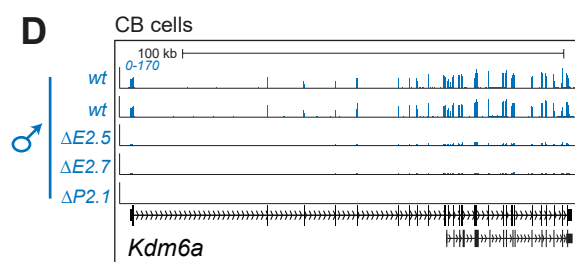**E**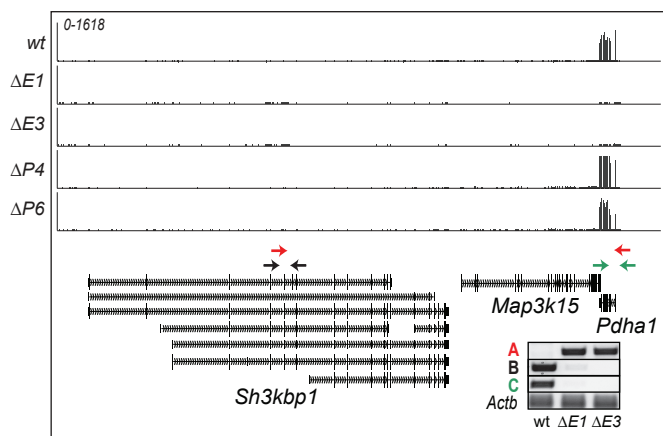

Supplement: Supplementary file 1 — Additional file 1: Fig. S1. CRISPR/Cas9 editing of Kdm6a in BC and CB mouse ES cells. (A) Schematic shows location of the exonic deletion (Kdm6aΔE) that removed exons 2–4 of Kdm6a in male BC and CB ES cells. Exons are shown as vertical bars with location of the PCR and RT-PCR primers (color-coded arrows) used to confirm the deletion and measure expression. Above, images of gels after electrophoresis of PCR products (gDNA), RT-PCR products (cDNA), and Western blot (protein) confirm KDM6A KO in male BC clones (Kdm6aΔE1, Kdm6aΔE3) and CB clones (Kdm6aΔE2.5, Kdm6aΔE2.7) compared to wt (color-coding refers to primers shown on schematic). Actb was run as a control for PCR and RT-PCR and Ponceau S staining was used as loading control for the Western blot. Sanger sequencing shown below the schematic was done to verify deletions in all male Kdm6aΔE clones compared to wt. (B) Schematic shows location of the promoter region deletion (Kdm6aΔP) made in male BC and CB ES cells. Exons are shown as vertical bars with location of the PCR and RT-PCR primers (color-coded arrows) used to confirm the deletion and measure expression. Above, images of gels after electrophoresis of PCR products (gDNA) and RT-PCR products (cDNA) confirm KDM6A KO in male BC clone (Kdm6aΔP4) and CB clone (Kdm6aΔP2.1) compared to wt. Actb was run as a control for PCR and RT-PCR. Sanger sequencing shown below the schematic was done to verify deletions in all Kdm6aΔP male BC and CB clones. (C-D) UCSC genome browser (GRCm38/mm10) view of RNA-seq profiles for (C) BC male and female wt, BC male clones Kdm6aΔE and Kdm6aΔP, and (D) CB male wt, and CB male Kdm6aΔE and Kdm6aΔP clones. Note that a low level of 3’ end reads are present in clones with deletion of exons 2–4, although there is no evidence of protein (see A above). In clones with deletion of the promoter, there are no reads over Kdm6a, including no reads overlapping the small alternative Kdm6a transcript, confirming absence of expression and suggesting the a [file 13293_2022_452_MOESM1_ESM.pdf]

Figure S2

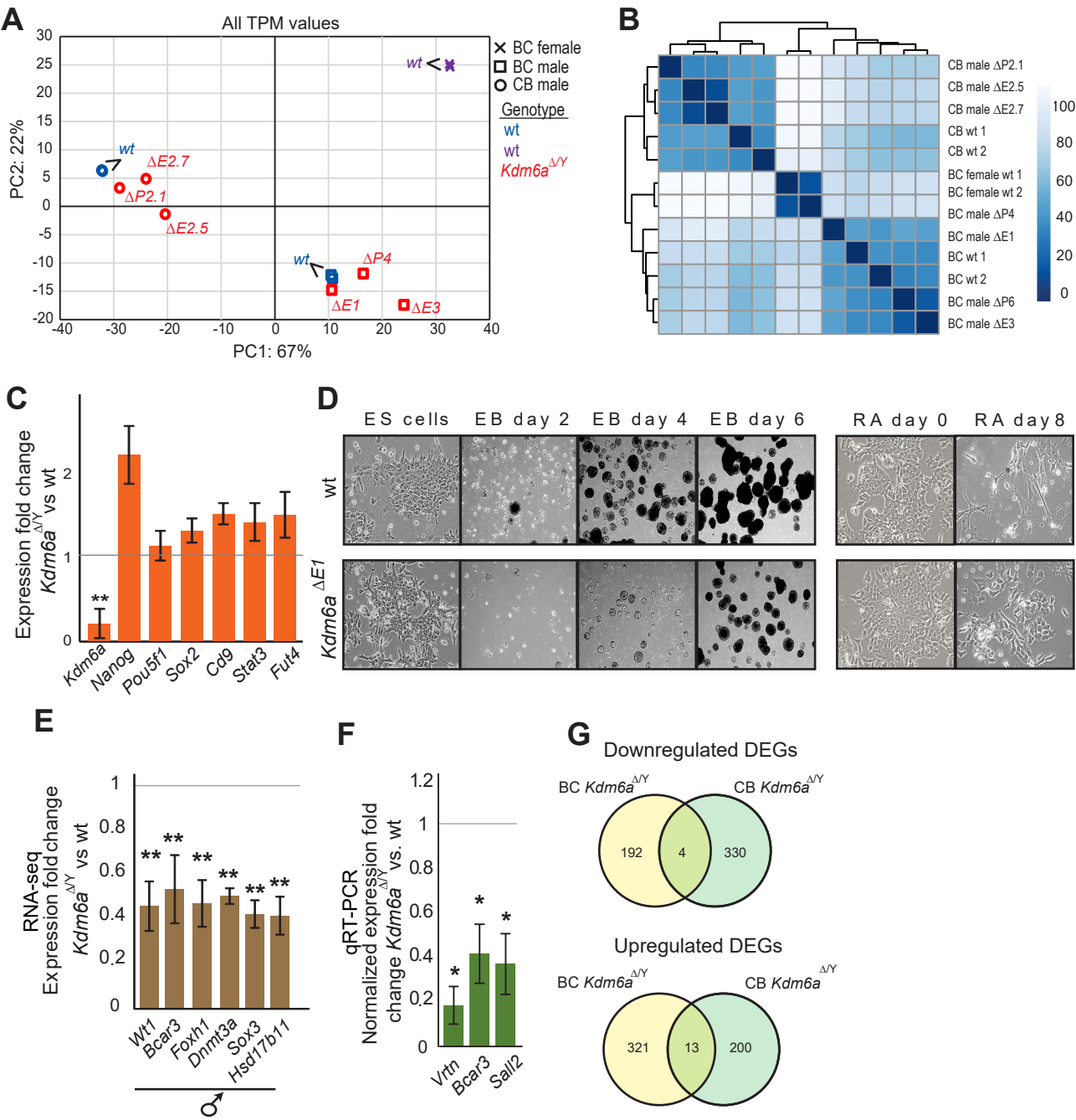

Supplement: Supplementary file 2 — Additional file 2: Fig. S2. Diploid gene expression changes in Kdm6a KO BC and CB clones. (A) Principal component analysis (PCA) based on diploid expression values for all transcribed genes from RNA-seq in wt and Kdm6a KO clones derived from the BC and CB crosses. BC clones include two male wt, two female wt, three male KO (Kdm6aΔ/Y), while CB clones include two male wt and three male KO (Kdm6aΔ/Y). Clone identifiers are included in the plot. (B) Hierarchal clustering of the clones described in (A). The color scale represents the sample-to-sample distance. (C) Expression fold changes between BC derived Kdm6aΔ/Y clones (Kdm6aΔE1, Kdm6aΔE3, and Kdm6aΔP4) and wt measured by RNA-seq show a significant decrease in Kdm6a expression, but no significant decrease in expression of pluripotent genes (Nanog, Pou5f1, Sox2, Cd9, Stat3, Fut4) (**p < 0.01 using a student’s t-test). (D) Deficiencies in ES cell differentiation potential following Kdm6a KO were tested by removal of LIF and by all-trans retinoic acid (RA) treatment. Smaller and less dense embryoid bodies were observed 6 days after removal of LIF in Kdm6aΔE1. In the presence of RA, wt cells show morphological signs of differentiation after 8 days, while Kdm6aΔE1 cells remain similar in morphology to day 0 controls. (E) Expression fold changes between male BC Kdm6aΔ/Y clones (Kdm6aΔE1, Kdm6aΔE3, and Kdm6aΔP4) and wt measured by RNA-seq confirm decreased expression of known KDM6A target genes Wt1, Bcar3, Foxh1, Dnmt3a, Sox3, Hsd17b11. Expression is based on diploid analysis at **FDR < 0.001. (F) Expression fold changes between male BC Kdm6aΔ/Y clones (Kdm6aΔE1, Kdm6aΔE3, and Kdm6aΔP4) and wt measured by quantitative RT-PCR analysis confirm downregulation of Vrtn, Bcar3, and Sall2 (*p < 0.01 using a student’s t-test) (Additional file 9A). Expression is normalized to Actb. (G) Venn diagrams to compare the number of downregulated and upregulated DEGs in male BC Kdm6aΔ/Y clones (Kdm6aΔE1, Kdm6aΔE3, and Kdm6aΔP4) and male CB K [file 13293_2022_452_MOESM2_ESM.pdf]

Figure S3

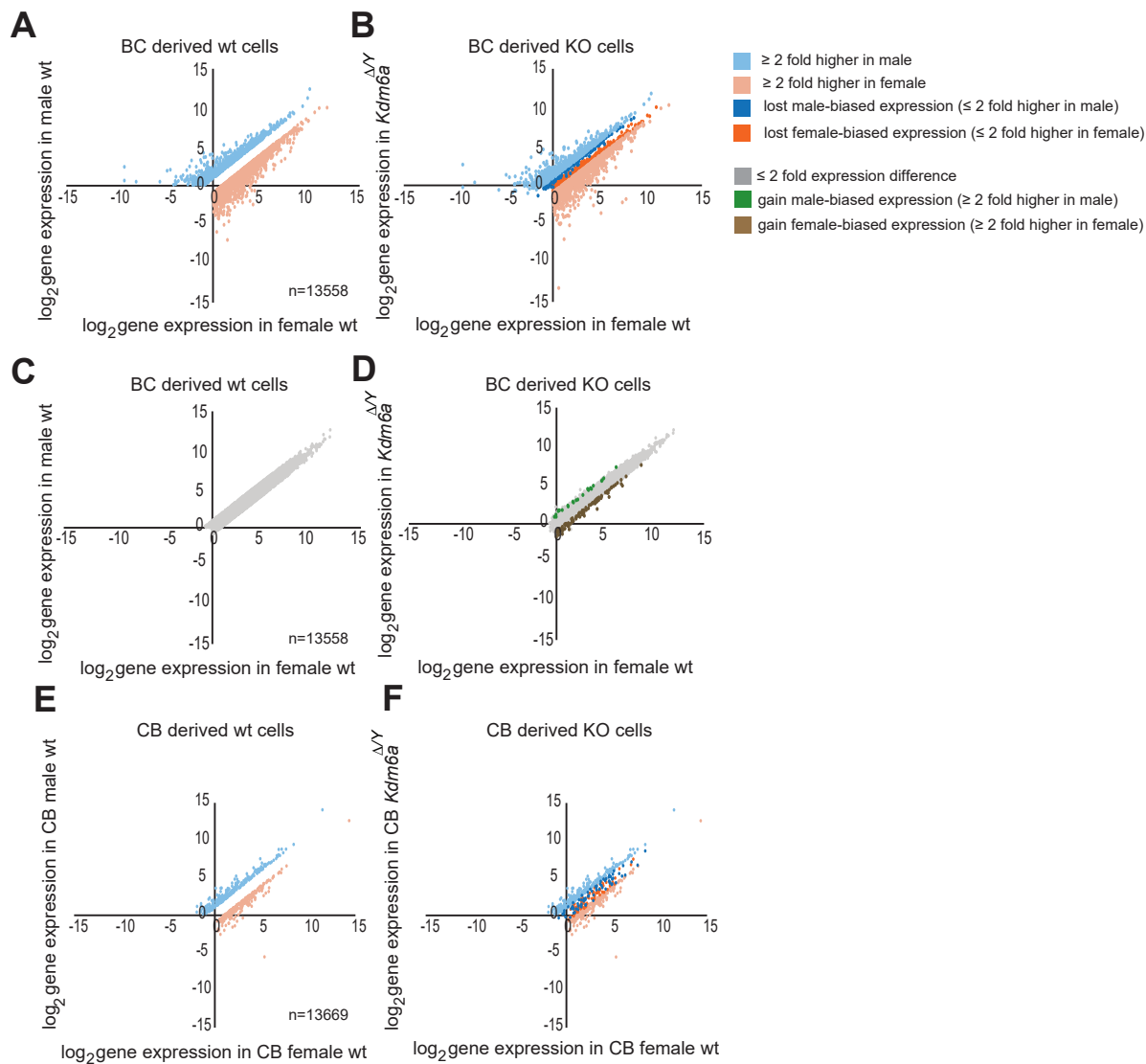

Supplement: Supplementary file 3 — Additional file 3: Fig. S3. Changes in sex-biased gene expression in Kdm6a KO BC and CB clones. (A) Scatter plots of log2 gene expression between male and female lines illustrating the degree of sex-biased expression in wt cells. Sex-biased genes were identified by comparing two BC male wt clones versus two BC female male wt clones. A gene was classified as sex-biased if its expression is ≥ 2 TPM fold expression higher in either female wt or male wt cells (p ≤ 0.05). Female-biased genes are in light orange and male-biased genes in light blue. (B) Scatter plot showing loss of sex-biased expression in three BC male Kdm6aΔ/Y clones (Kdm6aΔE1, Kdm6aΔE3, and Kdm6aΔP4) versus two BC female wt clones. Genes that lost sex-biased expression in KO cells are in dark orange if female-biased in wt, and in dark blue if male-biased in wt. (C) Scatter plot of genes without sex-biased expression (< 2 TPM fold expression difference) in wt BC cells. (D) Gain of sex biased expression in in three BC male Kdm6aΔ/Y clones (Kdm6aΔE1, Kdm6aΔE3, and Kdm6aΔP4). (E, F). Scatter plots of log2 gene expression of sex-biased genes in (E) three CB male wt clones versus three CB female wt clones, (F) three CB male Kdm6aΔ/Y (Kdm6aΔE2.5, Kdm6aΔE2.7, and Kdm6aΔP2.1) clones versus three CB female wt clones. Same analysis as in A, B. All wt expression values are from re-analysis of published RNA-seq data in CB ES cells, as we did not have access to a female wt CB line [34]. See also Table 1 and Additional file 10: Table S10. [file 13293_2022_452_MOESM3_ESM.pdf]

Figure S4

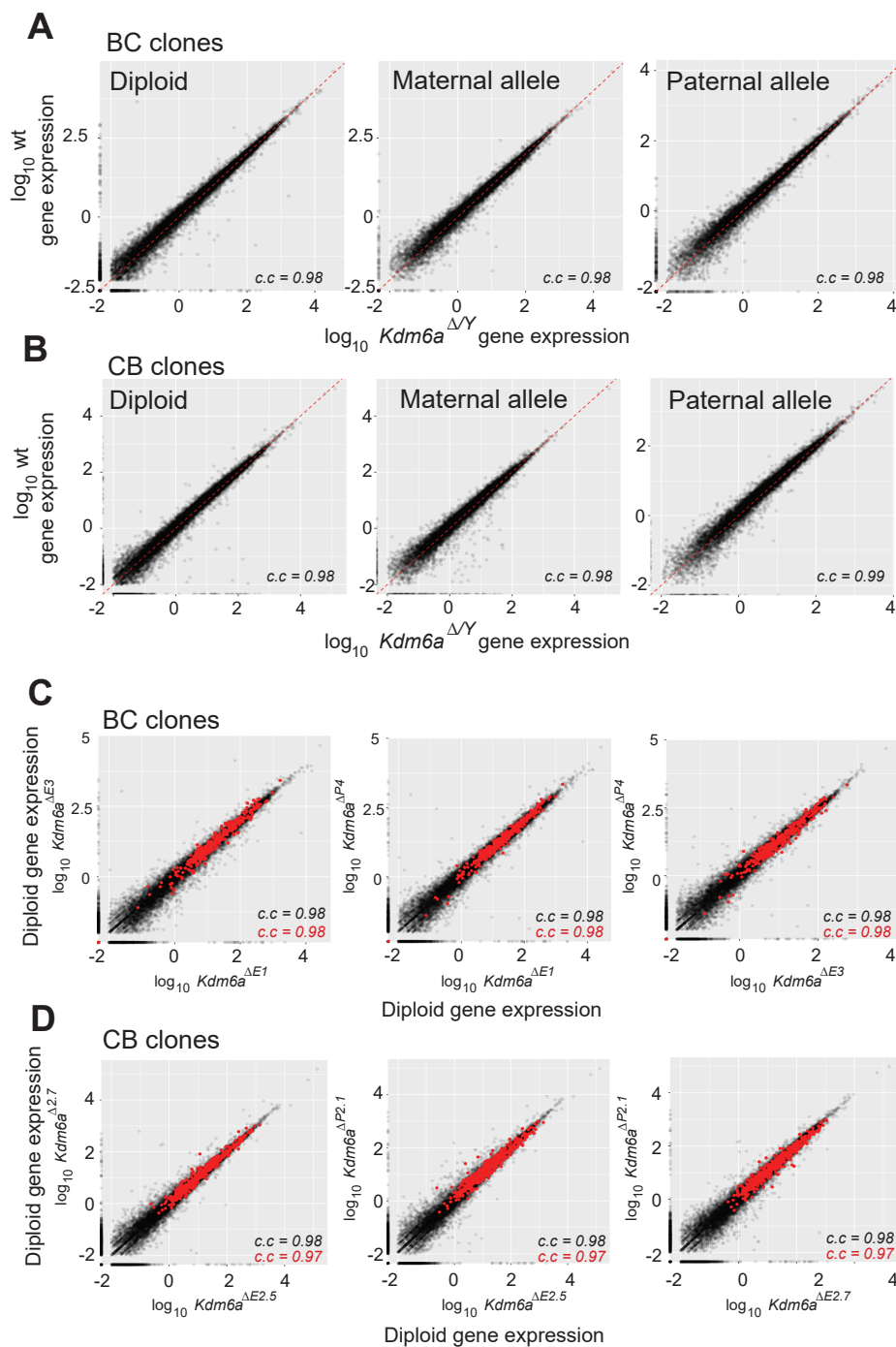

Supplement: Supplementary file 4 — Additional file 4: Fig. S4. Correlation of gene expression (diploid, maternal and paternal alleles) between wt and KO clones. Scatter plots of average gene expression based on TPM values show a high correlation between male BC wt and (A) Kdm6aΔ/Y clones (Kdm6aΔE1, Kdm6aΔE3, and Kdm6aΔP4), and (B) male CB Kdm6aΔ/Y clones (Kdm6aΔE2.5, Kdm6aΔE2.7, and Kdm6aΔP2.1) for diploid and allele-specific expression. Correlation coefficients are all ≥ 0.96. (C, D) Scatter plots of diploid TPM expression values comparing individual BC KO clones (C) and CB KO clones (D). Red dots represent all allelicly regulated genes (combined groups A-F). Black dots represent all other genes. High correlation coefficients (c.c) indicate similar expression changes following KO in all three clones. [file 13293_2022_452_MOESM4_ESM.pdf]

Figure S5

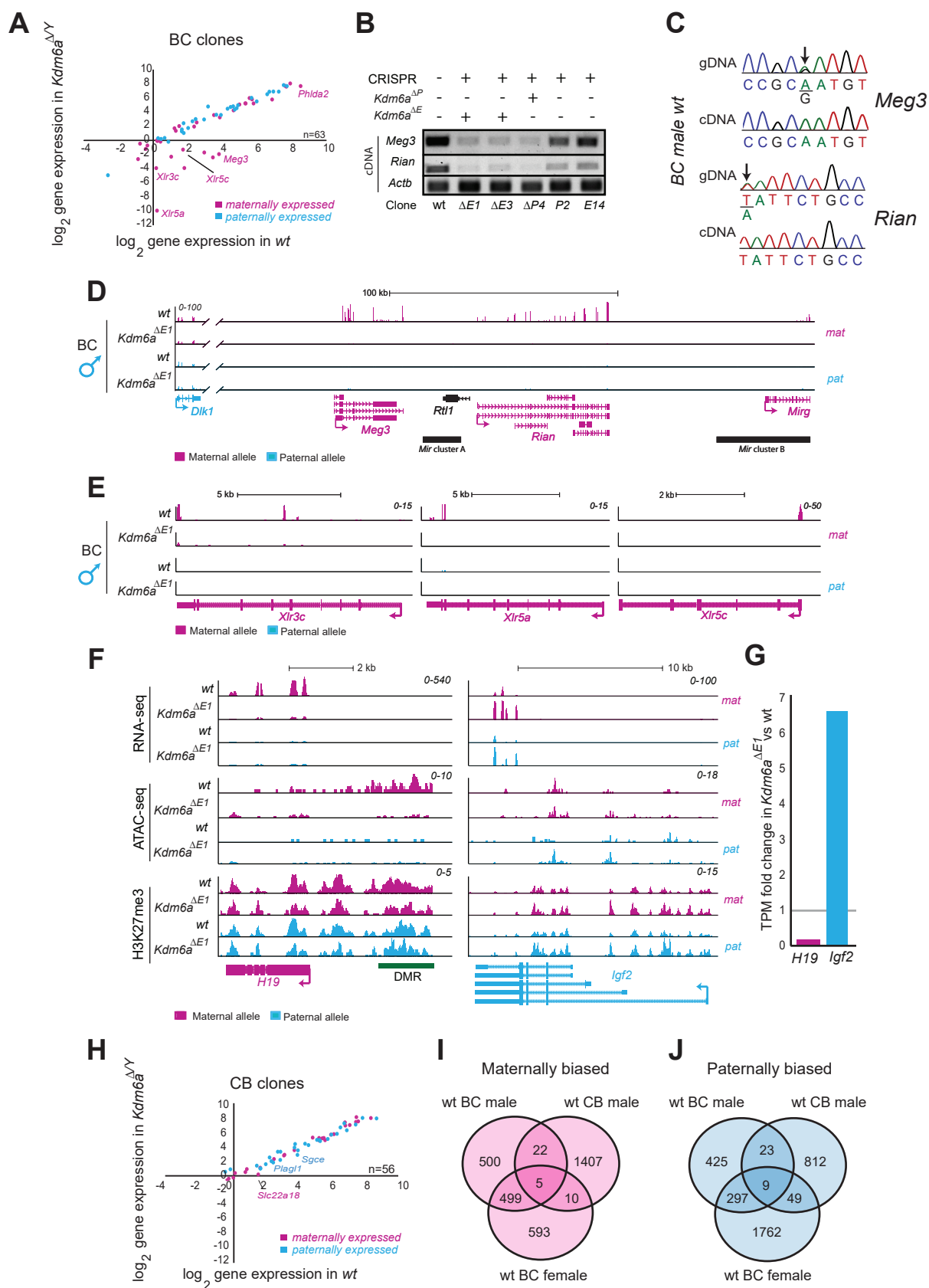

Supplement: Supplementary file 5 — Additional file 5: Fig. S5. A subset of maternally expressed imprinted genes are downregulated after Kdm6a KO in BC but not in CB clones. (A) Scatter plot of average expression changes for canonical imprinted genes, either maternally (purple) or paternally (blue) expressed, in Kdm6aΔ/Y (Kdm6aΔE1, Kdm6aΔE3, and Kdm6aΔP4) versus wt (average from 2 clones) based on diploid RNA-seq analysis in male BC derived clones. Only imprinted genes with > 1TPM in at least one sample are shown. (B) RT-PCR analysis confirms decreased expression of Meg3 and Rian in all male BC KO clones (Kdm6aΔE1, Kdm6aΔE3, Kdm6aΔP4). CRISPR + /– labels indicate presence/absence of transfection with CRISPR/Cas9 and Kdm6a sgRNAs, while a ± for Kdm6aΔP and Kdm6aΔE clones indicates editing result. Non-edited CRISPR control clones (Kdm6aE14, Kdm6aP2) maintain expression of these genes. (C) Sanger sequencing in wt male BC clones confirmed the presence of SNPs (gDNA) and mono-allelic expression (cDNA) of Meg3 and Rian. (D, E) UCSC Genome browser (GRCm38/mm10) views of allele-specific RNA-seq profiles at the maternally expressed Dlk1/Mirg polycistron region and at a subset of imprinted Xlr genes. Allelic read profiles on the maternal chromosome (purple) and the paternal chromosome (blue) are based on SNP analysis in male BC wt clone (KO-), compared to male BC Kdm6aΔE1 clone (KO +). (F) UCSC Genome browser (GRCm38/mm10) view of allele-specific RNA-seq, ATAC-seq and H3K27me3 ChIP-seq profiles generated in male wt and Kdm6aΔE1 at the imprinted genes H19 (maternally expressed) and Igf2 (paternally expressed). The DMR (differentially methylated region) is denoted by a green bar. (G) TPM expression fold change measured by RNA-seq for H19 and Igf2 in Kdm6AΔE1 versus wt shows H19 downregulation concordant to the expected upregulation of Igf2. (H) Same analysis as in (A) for male CB Kdm6a Δ/Y clones (Kdm6aΔE2.5, Kdm6aΔE2.7, and Kdm6aΔP2.1). (I, J) Venn diagrams to compare the number of allele-biased genes in BC male [file 13293_2022_452_MOESM5_ESM.pdf]

Figure S6

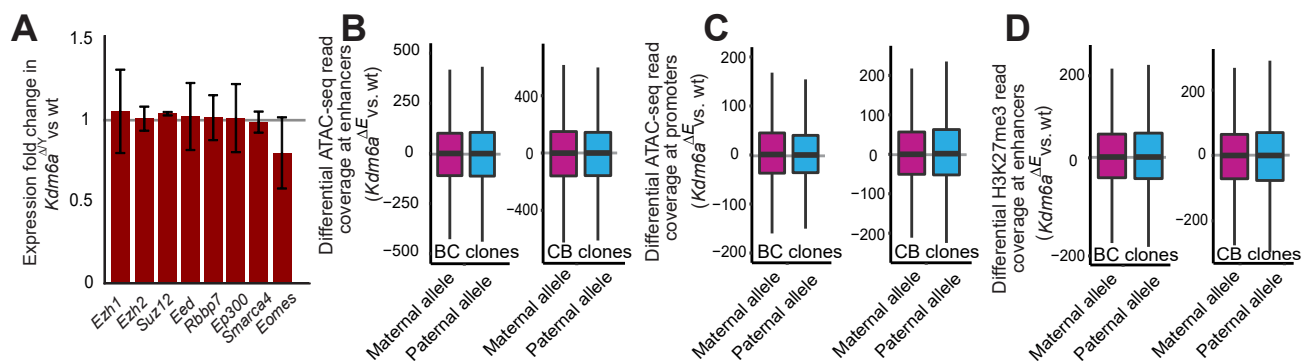

Supplement: Supplementary file 6 — Additional file 6: Fig. S6. Chromatin modifier expression and chromatin accessibility changes at enhancers and promoters after Kdm6a KO. (A) Expression fold changes between Kdm6aΔ/Y clones (Kdm6aΔE1, Kdm6aΔE3, and Kdm6aΔP4) and wt measured by RNA-seq for genes encoding known chromatin modifying proteins in the PRC2 complex (Ezh1/2, Suz12, Eed, Rbbp7) and the MLL complex (Ep300, Smarca4, Eomes). (B) Box plots of allelic ATAC-seq read coverage at gene enhancers in male BC and CB clones (Kdm6aΔE1 and Kdm6aΔE2.5) compared to their respective wt controls. Enhancers (n = 25,346) were defined as regions enriched in H3K4me3 and H3K27ac in male ES cells as described [12]. (C) Same analysis as in (B) but for promoters (n = 20,745). ATAC-seq coverage was calculated using ± 2 kb surrounding the transcription start sites determined using GENCODE. No significant chromatin accessibility differences were seen, nor was there a significant difference between alleles in either cross. (D) Same analysis as is (B) but for H3K27me3. [file 13293_2022_452_MOESM6_ESM.pdf]

Figure S7

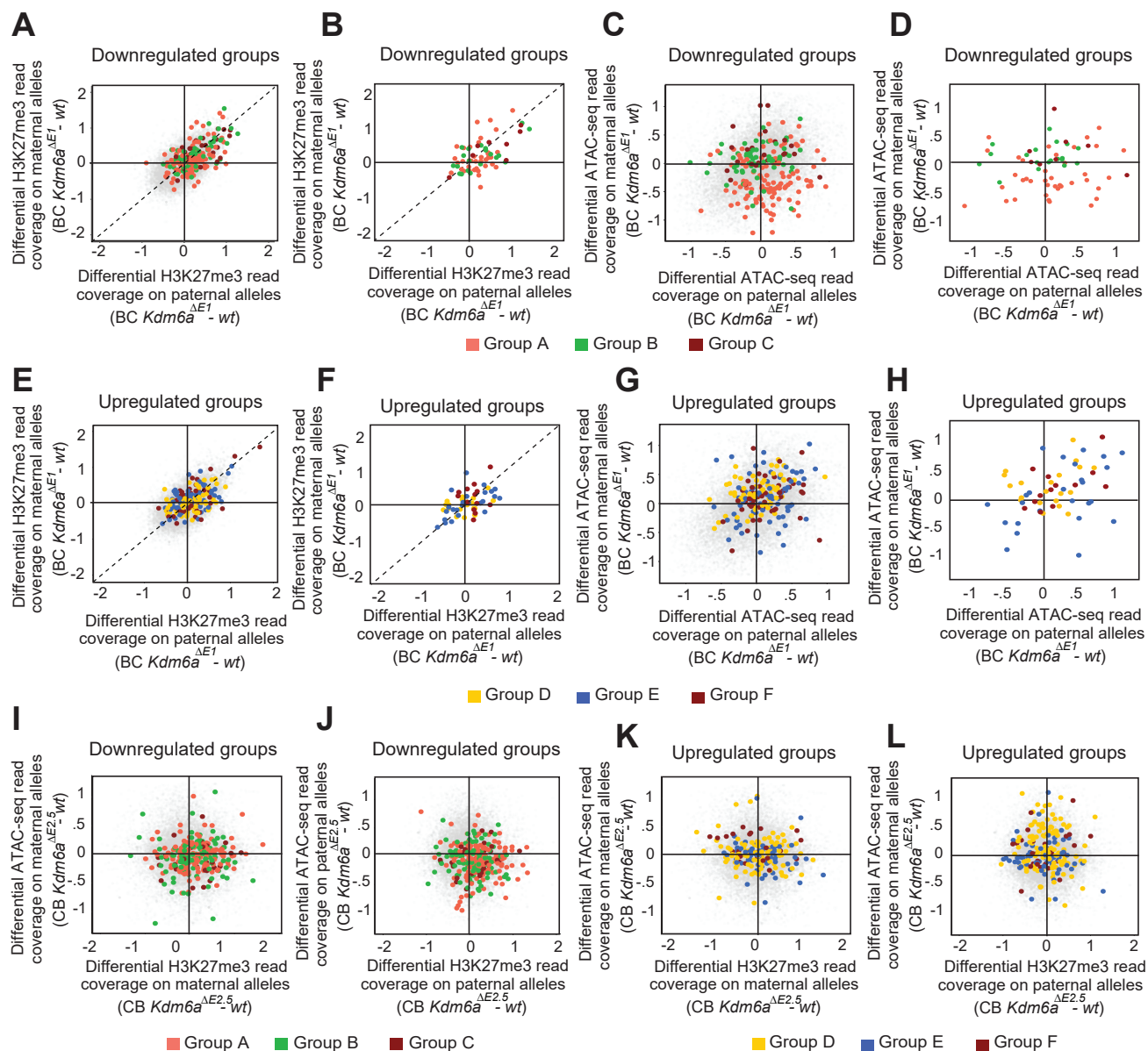

Supplement: Supplementary file 7 — Additional file 7: Fig. S7. Allelic epigenetic features of genes regulated by KDM6A. (A) Scatter plots of H3K27me3 read coverage in log2 scale on maternal and paternal alleles in male BC Kdm6aΔE1 cells versus wt. Allele-specific ChIP-seq reads around promoters (± 2 kb of the TSS) were used to calculate promoter coverage normalized by sequencing depth and allele differences (see also Fig. 5A, D). (B) Same analysis as in (A) but for DEGs that overlap between exonic KO clones only (Kdm6aΔE1 and Kdm6aΔE3) and Kdm6aΔY clones (Kdm6aΔE1, Kdm6aΔE3 and Kdm6aΔP4). (C) Scatter plots of ATAC-seq read coverage in log2 scale on maternal and paternal alleles in male BC Kdm6aΔE1 cells versus wt. Allele-specific reads around promoters (± 2 kb of the TSS) were used to calculate promoter coverage normalized by sequencing depth and allele differences (see also Fig. 5B, E). (D) Same analysis as in (C) but for DEGs that overlap between exonic KO clones only (Kdm6aΔE1 and Kdm6aΔE3) and Kdm6aΔY clones (Kdm6aΔE1, Kdm6aΔE3 and Kdm6aΔP4). (E, F) Same analysis as in (A) and (B), but for upregulated DEGs. (G, H) same analysis as in (C) and (D), but for upregulated DEGs. (I, J) Scatter plots of ratios of ATAC-seq read coverage versus ratios of H3K27me3 ChIP-seq read coverage between CB clone Kdm6aΔE2.5 and wt CB cells at promoter regions (± 2 kb of the TSS) of downregulated groups on maternal and paternal alleles. (K, L) Same analysis as in (I) and (J) but for upregulated groups. See also Fig. 5G, H. Unedited image figure legend. (A) Raw images for electrophoresis gels shown in Additional file 1A for BC and CB clones. Left panel shows screening of CRISPR exon deletion clones by PCR. The top right gel shows screening of CRISPR exon deletion clones in CB cells by PCR (gDNA). The associated control Actb lanes are in (C) lower right gel (gDNA). For all panels, primer names correspond to those in Additional file 1A. (B) Unedited images of western blots and controls for KDM6A. Top two are film images [file 13293_2022_452_MOESM7_ESM.pdf]
